# Comparative Venomics of the *Vipera ammodytes transcaucasiana* and *Vipera ammodytes montandoni* from Turkey Provides Insights into Kinship

**DOI:** 10.3390/toxins10010023

**Published:** 2018-01-01

**Authors:** Benjamin-Florian Hempel, Maik Damm, Bayram Göçmen, Mert Karis, Mehmet Anıl Oguz, Ayse Nalbantsoy, Roderich D. Süssmuth

**Affiliations:** 1 Department of Chemistry, Technische Universität Berlin, 10623 Berlin, Germany; benjamin.hempel@chem.tu-berlin.de (B.-F.H.); maik.damm@fu-berlin.de (M.D.); 2 Department of Biology, Ege University, 35100 Izmir, Turkey; cypriensis@yahoo.com (B.G.); mert.karis@hotmail.com (M. K.); an_oguz@hotmail.com (M.A.O.); 3 Department of Bioengineering, Ege University, 35100 Izmir, Turkey

**Keywords:** *Viperidae*, *Vipera ammodytes*, Transcaucasian Nose-horned Viper, Transdanubian Sand Viper, snake venomics, intact mass profiling, tripeptide metalloprotease inhibitor, cytotoxicity, The detailed characterization and comparison of the venom proteome *Vipera ammodytes transcaucasiana* and *Vipera ammodytes montandoni* showed an impressive matching of the venom composition, which could help to overcome the question of the taxonomic status of *Vipera ammodytestranscaucasiana.* Additional bioactivity screenings against several cancerous and non-cancerous cell lines showed promising results against breast cancer cells.

## Abstract

The Nose-horned Viper (*Vipera ammodytes*) is one of the most widespread and venomous snakes in Europe, which causes high frequent snakebite accidents. The first comprehensive venom characterization of the regional endemic Transcaucasian Nose-horned Viper (*Vipera ammodytes transcaucasiana*) and the Transdanubian Sand Viper (*Vipera ammodytes montandoni*) is reported employing a combination of intact mass profiling and bottom-up proteomics. The bottom-up analysis of both subspecies identified the major snake protein families of viper venoms. Furthermore, intact mass profiling revealed the presence of two tripeptidic metalloprotease inhibitors and their precursors. While previous reports applied multivariate analysis techniques to clarify the taxonomic status of the subspecies, an accurate classification of *Vipera ammodytes* *transcaucasiana* is still part of the ongoing research. The comparative analysis of the viper venoms on the proteome level reveals a close relationship between the *Vipera ammodytes* subspecies, which could be considered to clarify the classification of the Transcaucasian Nose-horned Viper. However, the slightly different ratio of some venom components could be indicating interspecific variations of the two studied subspecies or intraspecies alternations based on small sample size. Additionally, we performed a bioactivity screening with the crude venoms against several human cancerous and non-cancerous cell lines, which showed interesting results against a human breast adenocarcinoma epithelial cell line. Several fractions of *Vipera a. transcaucasiana* demonstrated a strong cytotoxic effect on triple negative MDA MB 231 breast cancer cells.

## 1. Introduction

Venom research has an ongoing significance for various disciplines and applications ranging from drug development, pharmacology for rational antivenom production even to the cosmetics industry [1,2,3]. After the discovery of the first venom-derived therapeutic, Captopril, in 1975, which was developed from the Brazilian pit viper (*Bothrops jararaca*), in the following years, investigations of several other venomous snakes revealed further venom-based drugs with different medical applications [4,5]. Nevertheless, there is still an uncountable number of venomous animals in diverse habitats to be suspected, of which only a small part of venom proteomes have been characterized, and as a consequence the potential for new applications of these venoms and individual components thereof still have to be explored [3,6,7].

The advances in “-omics” technologies allowed for the characterization of an increasing number of animal venoms and plays a pivotal role for the development of new potential drugs against several human diseases. The increase in sensitivity and the development of soft ionization methods, e.g., electrospray ionization (ESI) or matrix-assisted laser desorption/ionization (MALDI), for mass spectrometry and next-generation high-throughput sequencing dramatically enhanced the analysis of venoms [3,8,9,10]. Nowadays, there exist different well-established protocols to characterize the venom in its entirety. The so-called bottom-up strategies can be divided in gel-based approaches and liquid chromatographic (LC)-based approaches. A combination of both strategies, termed “snake venomics”, uses both separation techniques successively followed by an in-gel digestion of excised protein bands and a mass spectrometric measurement by tandem mass analysis [11,12,13]. Recently described methods for the proteomic analysis of snake venoms include the “intact mass profiling”, which directly separate the components out of the crude venom without any previous fractionation. The power of this tool has already been demonstrated at the example of whole snake venom proteomes [14,15]. The intact mass analysis of native proteins compared to chemically reduced proteins allows for a classification, based on the number of existing intra- and intermolecular disulfide bonds, and represents an important characteristic of different viper-venom protein families [16,17,18]. However, the method is critical for proteins of higher molecular masses, e.g., snake venom metalloproteases, because the high resolution of accurate isotopic masses becomes challenging [15]. The subsequent step to the intact mass profiling is the MS/MS analysis by collision-induced dissociation (CID) of intact molecular masses, termed “top-down venomics”. The major advantage of this approach is a high-throughput analysis of venoms without the necessity of further pre-MS separation steps. A decisive drawback is the application mainly limited to peptides and small-sized proteins (>15 kDa), which are the main constituents of *Elapidae* (e.g., three-finger toxins, Kunitz-type inhibitor, etc.) [19,20,21]. In contrast, *Viperidae* venoms contain higher molecular mass components that makes them less suitable for the top-down approach as *Elapidae* venoms [14,15]. Finally, the mass spectrometry-based absolute intact mass quantification by isotope dilution is a further cutting-edge approach, which could replace the semi-quantitative densitometric determination [17,22,23].

The combination of several workflows allows for an encompassing characterization of different kinds of venoms. In particular, the venom of vipers is a promising source of new substances and therapeutics, due to their different venom compositions [1,4]. They are distributed in a wide range all over the world, and are especially located around the Mediterranean Sea [24]. A great variety of habitats and zones of subtropical climate along the north coast side of Turkey provides suitable places to shelter for many species that belong to the *Viperidae* family [25,26]. Important major protein families found in analyzed viperid venoms are snake venom metalloproteases (svMP), snake venom serine proteases (svSP), hyaluronidases, 5′-nucleotidase, phospholipases A_2_ (PLA_2_), disintegrins, C-type lectin like proteins (CTL), cysteine-rich secretory proteins (CRISP), natriuretic peptides, bradykinin-potentiating peptides (BPP), nerve growth factors (NGF), snake venom vascular endothelial growth factors (VEGF-F) and Kunitz-type protease inhibitors [27,28].

Our ongoing studies on snake venoms focus on the venom characterization of unrecorded *Vipera* in the Turkish area and initial cytotoxicity screenings against cancerous as well as non-cancerous cell lines of potent bioactive peptides and proteins. From this point of view, we aimed to screen viper venoms from different regions of Turkey. For this purpose, the regional endemic Transcaucasian Nose-horned Viper (*Vipera ammodytes transcaucasiana*) from central Anatolia and the *V. a. montandoni* from Northwest of Turkey (Turkish Thrace) were chosen for a comparative venom investigation. The Nose-horned Viper (*Vipera ammodytes*) is one of the most venomous snakes in Europe and common pathophysiological conditions range from local tissue damage, hemorrhage, pain, paralysis up to necrosis and in some cases even death [29,30]. After the description of *Vipera ammodytes ammodytes* (Linnaeus, 1758), five further subspecies have been described: *V. a. meridionalis* [31], *V. a. montandoni* [32], *V. a. transcaucasiana* [33], *V. a. ruffoi* [34] and *V. a. gregorwallneri* [35]. *V. a. transcaucasiana* is considered a separate species by some authors [36]. Heckes et al. (2005) and Tomovic (2006) accepted only four valid taxa for *V. ammodytes* (*V. a. ammodytes*, *V. a. meridionalis*, *V. a. montandoni* and *V. a. transcaucasiana*) with an extensive investigation on the species [37,38]. Phylogenetic and phylogeographic studies, using mtDNA gene sequences obtained from cytochrome *b* (cyt *b*), 16S rRNA and the noncoding control region, supported the validation of subspecies status of *V. a. ammodytes*, *V. a. meridionalis* and *V. a. montandoni*, but in turn *V. a. ruffoi* and *V. a. gregorwallneri* were only accepted as synonyms to the nominotypic subspecies, *V. a. ammodytes*. In addition, the taxonomic status of *V. a. transcaucasiana* was tentatively classified as subspecies due to a low sample size [39].

**Figure 1:**
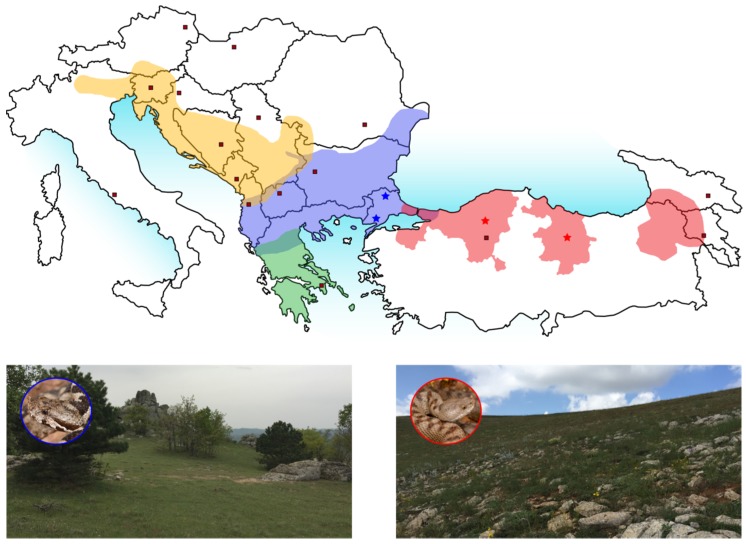
Geographical distribution of subspecies from *Vipera ammodytes.* The distribution areas of the four *Vipera ammodytes* subspecies are highlighted in color: *V. a. ammodytes* (yellow), *V. a. montandoni* (blue), *V. a. meridionalis* (green) and *V. a. transcaucasiana* (red). Overlapping distribution areas are highlighted by shaded colors. The locations for catches of *V. a. montandoni* (star, blue) and *V. a. transcaucasiana* (star, red) are marked and exemplary snake habitats are shown.

The occurrence of *Vipera ammodytes* distributes around the Mediterranean Sea and reaches from the Alps over to Turkey, Georgia, Azerbaijan and Iran. The Transcaucasian Nose-horned Viper (*Vipera ammodytes transcaucasiana* (Vat)) shows a distribution in the Northeast of Turkey and sections of Georgia along the Black Sea coast and some inland provinces in Turkey (see Figure 1, red) [37,40]. The Transdanubian Sand Viper (*Vipera ammodytes montandoni* (Vam)) is spread from Turkish Thrace, Bulgaria to Romania and shares its distribution area in parts with all three other subspecies (see Figure 1, blue) [37]. Beside those previously mentioned, there exist two further subspecies whose venoms were already characterized: The Western Sand Viper (*Vipera ammodytes ammodytes*) can be found from the Alps over Croatia to the borders of Macedonia (see Figure 1, yellow) and the Eastern Sand Viper (*Vipera ammodytes meridionalis*), only endemic in Greece and several Hellenic islands (see Figure 1, green) [41]. All subspecies of *Vipera ammodytes* can be found from sea level up to 2000 m a.s.l. in many kinds of suitable habitats (forests, meadows, arid regions, rocky areas, and even sandy coastal parts), thus there is no special habitat selectivity. The Nose-horned viper (*Vipera ammodytes*) is one of the most and venomous species in Europe and therefore of significance for public health [41,42].

Previous investigations on the neutralization of lethality by several antisera against *Vipera ammodytes* subspecies revealed low paraspecific neutralization potency [43,44]. Therefore, the elucidation of the undescribed venom proteome is significant for public health and could help to bypass the lack of sufficient venom neutralization. Here, we give deeper insight into the composition of the venom proteome and peptidome of the two Nose-Horned vipers by bottom-up venomics and an intact mass profiling of the crude venoms. The detailed characterization and comparison of the venom proteomes with other subspecies showed a remarkable matching of the venom components, which could be an additional helpful tool to overcome the controversial question of the taxonomic status of *Vipera ammodytes transcaucasiana* in connection with the phylogentic analysis [39]*.*

## 2. Results and Discussion

### 2.1. The Venom Proteome

#### 2.1.1. Intact Mass Profiling

**Figure 2:**
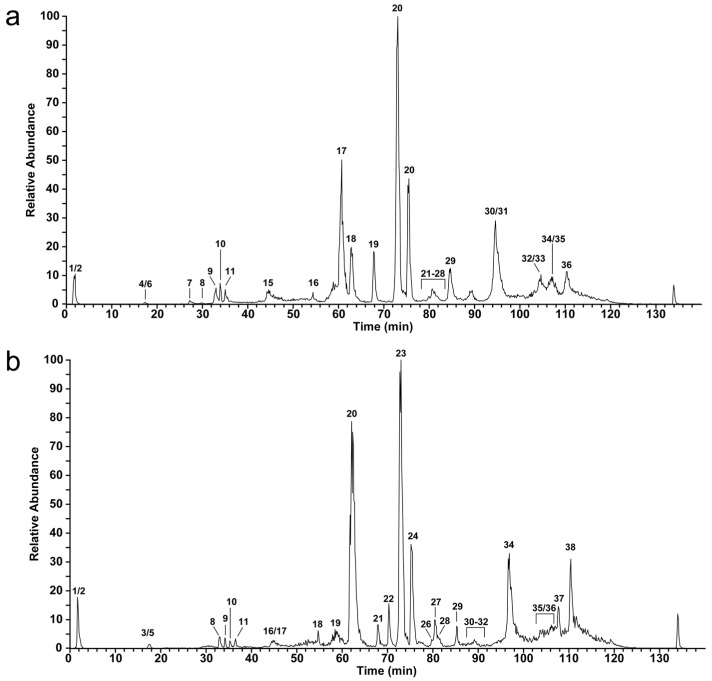
Intact molecular mass profiles of *V. a. transcaucasiana* (Vat) and *V. a. montandoni*. (Vam). The total ion counts (TIC) of crude venoms from: (**a**) Vat; and (**b**) Vam were measured by HPLC-ESI-MS. The peak nomenclature is based on the chromatogram fractions and is shown in Figure 3. The identified molecular masses of intact proteins and peptides are listed for Vat in Table S1 and Vam in Table S2.

First, we applied an intact venom molecular mass profiling to obtain an overview of molecular masses from all venom components, including low abundant and low molecular mass compounds. Therefore, the crude venom as well as the RP-HPLC separated peptide fractions were used. The initial profiling of *Vipera ammodytes transcaucasiana* (Vat) revealed 117 molecular masses for different venom components (see Figure 2a, Table 1 and Table S1): 55 (<1 kDa), 11 (1–3 kDa) and 28 (3–9 kDa), which represents the peptide part of the venom in total with 13.49%. Higher molecular masses from 10 to 28 kDa were detected 23 times in the following composition: 1 (10 kDa), 14 (12–16 kDa), 2 (21 kDa) and 6 (25–28 kDa). The venom of *Vipera ammodytes montandoni* (Vam) showed a comparable distribution pattern with 115 different venom components (see Figure 2b, Table 1 and Tables S2): 47 (<1 kDa), 26 (1–3 kDa) and 19 (3–8 kDa), which corresponds to a slightly higher peptide content of 17.49%. We also found 25 components with molecular masses between 13 and 34 kDa: 10 (13–16 kDa), 2 (21 kDa), 7 (24–27 kDa) and 6 (32 kDa).

Furthermore, the initial mass profiling enabled us to identify two peptides as members of the tripeptide metalloprotease inhibitor (svMP-i) family. The protease inhibitors serve to avoiding damages during storage of the venom in the gland tissue as well as to prevent autoproteolysis of the venom. Protective effects have been described for the well-known endogenous pyroglutamic tripeptide metalloprotease inhibitors, e.g., the small tripeptides pEQW and pENW [45]. Their strong inhibitory effect against svMP from different vipers was intensively studied [46,47,48]. In our studies, the venoms of *V. a. transcaucasiana* and *V. a. montandoni* exhibit two molecular masses with *m*/*z* 444.23 and *m*/*z* 430.17 (see Figure 4 and Tables S1 and S2). While the molecular mass of *m*/*z* 444.23 corresponds to a well separated peptide signal found in the venoms of Vat (see Figure 2; Peak 4/6) and Vam (see Figure 2; Peak 3, 4/5), the intensity of the signal at *m*/*z* 430.17 for Vat (see Figure 2; Peak 7) and Vam (see Figure 2; Peak 6) is less prominent.

Accordingly, the measured mass of *m*/*z* 430.17 matches to the predicted monoisotopic mass of the pyroglutamic tripeptide pENW, while the measured *m*/*z* 444.23 matches to the predicted monoisotopic mass of the pyroglutamic tripeptide pEKW. The identities of the pyroglutamic tripeptides pEKW and pENW were ultimately confirmed by ESI-MS/MS experiments (see Figure 4). In addition, comparison with spectra for the pEKW inhibitor from the literature coincides with our experimentally observed fragmentation pattern [49]. As an additional evidence for tripeptide metalloprotease inhibitors in the venom of both vipers, the presence of several Gly- and Pro-rich protein fragments of the endogenous precursors were detected in the venoms of *V. a. transcaucasiana* as well as *V. a. montandoni* [50]. The peptide PEGPPLMEPHE, a previously described tripeptide precursor fragment [51] from the related snake *V. a. ammodytes*, was detected in both investigated venoms (see Figures S1 and S2 and Tables S1 and S2, Peak 9 and 8). Furthermore, several other precursor peptide fragments of this type were detected in the venoms of Vat (GGGGGGW, PPQMPGPKVPP) and Vam (DNEPPKKVPPN) (see Figures S3–S6 and Tables S1 and S2). Taken together, the metalloprotease inhibitors and their precursor peptides form the major share of the peptidome of Vat (28.80%) as well as of Vam (45.93%), with the tripeptide pEKW as the main component. Even if details on secretion and processing of the tripeptides are still unknown, a formation of the pyroglutamic inhibitors is assumed to happen during the exocytosis. A processing in the gland lumen itself is rather unlikely based on missing glutaminyl cyclases [49].

A drawback of the intact mass profiling is that high molecular masses become difficult to properly detect. A reason is that the higher charged states of molecular ions cause a high peak density which increasingly challenges the limits in resolution of the orbitrap mass analyzer. Hence, the incomplete characterization of the venoms discriminating higher masses requires the complementary bottom-up approach.

#### 2.1.2. Bottom-Up Venomics

**Figure 3:**
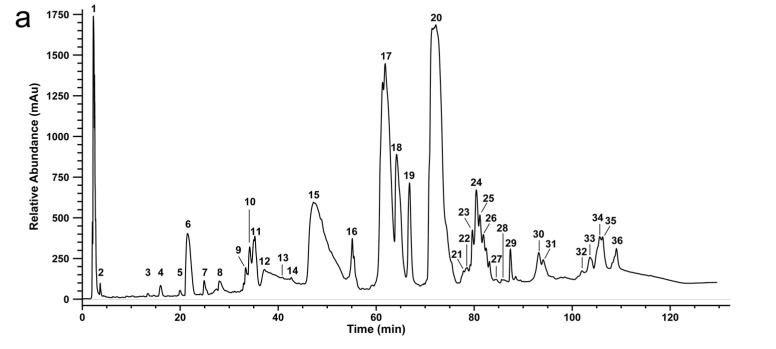

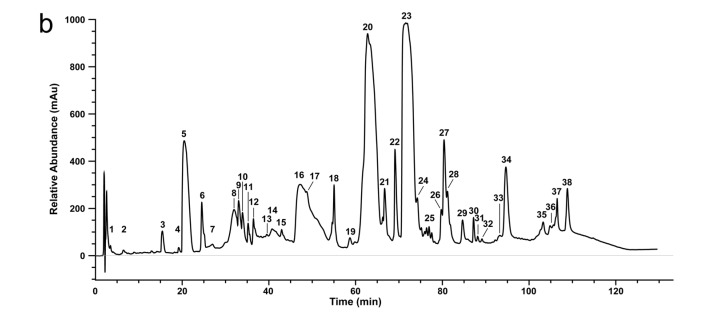
Chromatograms of *V. a. transcaucasiana* (Vat) and *V. a. montandoni*. (Vam) venoms separated by semi-preparative reversed-phase HPLC. Venom separation of: (**a**) Vat; and (**b**) Vam was performed by a Supelco Discovery BIO wide Pore C18-3 RP-HPLC column and UV absorbance measured at 214 nm.

The bottom-up analysis by the combined approach, termed snake venomics, was performed with lyophilized snake venoms of Vat and Vam. Subsequent to fractionation by reversed phase-HPLC (see Figure 3), the protein containing fractions were size-separated by SDS-PAGE (see Figure 5). The prominent bands were excised followed by tryptic in-gel digestion and *de novo* sequencing via MS/MS. The quantitative venom composition was calculated based on the RP-HPLC peak integration and in case of co-eluting components, the ratio of optical intensities and densities from SDS-PAGE was deduced [17,22,23].

A concluding analysis of the Vat and Vam venoms rendered the following results (see Figure 6): the most abundant toxin family of the Vat venom is represented by snake venom phospholipases A_2_ (svPLA_2_, 44.96%) followed by vascular endothelial growth factors (VEGF-F, 9.81%), snake venom serine proteases (svSP, 9.47%), snake venom metalloproteases (svMP, 8.76%), l-amino acid oxidases (LAAO, 6.41%), cysteine-rich secretory proteins (CRISP, 3.41%) and C-type lectin like proteins (CTL, 2.99%). The remaining constituents, such as a disintegrin and a phosphodiesterase, were summarized as other proteins (0.07%) and unannotated proteins (0.60%) (see Figure 6a), respectively. Similarly, the venom of Vam is composed of snake venom phospholipases A_2_ (svPLA_2_, 52.44%) as main part followed by vascular endothelial growth factors (VEGF-F, 10.69%), snake venom serine proteases (svSP, 5.48%), l-amino acid oxidases (LAAO, 4.83%), cysteine-rich secretory proteins (CRISP, 3.81%), snake venom metalloproteases (svMP, 1.79%), C-type lectin like proteins (CTL, 0.26%), several trypsin-like proteins and one aminopeptidase (other proteins, 0.11%) and non-characterized proteins (3.11%) (see Figure 6b).

In total, 118 fragments with 84 different sequences for Vat (see Table 1 and Tables S1) and 87 fragments to 66 sequences for Vam (see Table 1 and Tables S2) could be assigned by bottom-up analysis with a subsequent de novo sequencing. The assignment revealed, e.g., for the phospholipase A_2_ protein family, homologs of both chains from the heterodimeric Vaspin (acidic, Uniprot-ID: CAE47105.1; basic, Uniprot-ID: CAE47300.1), one of Vipoxin (B chain, Uniprot-ID: 1AOK_B) and the Ammodytin. This monomeric PLA_2_ was identified in the case of Vat as the neutral Ammodytin I2(A) variant (Uniprot-ID: CAE47197.1) and in the venom of Vam by two isoforms of the acidic Ammodytin I1(A) (Uniprot-ID: CAE47141.1) and I1(C) (Uniprot-ID: CAE47172.1). The main snake venom serine proteases (svSP) could be identified as homologs of Nikobin (Uniprot-ID: E5AJX2.1) and the Cadam10-svSP11 (Uniprot-ID: JAV48393.1). The identity of several bottom-up determined proteins, e.g., Vipoxin or Ammodytin, could be additionally assigned by intact mass profiling through comparison to their average protein family masses.

### 2.2. Comparative Venomics of Vipera ammodytes

Correlations between venom composition and relationship from snakes belonging to the same genera have been shown in the literature [52,53]. Nevertheless, many examples for interspecies variations in the composition of snake venoms are also described [54,55,56,57]. Variations in the venom composition can be associated to different diets, regional separation of populations, sex or age [58,59,60,61,62]. Likewise, Tashima et al. [63] reported significant variations in the venom composition of close-related pitvipers, but could identify taxonomy markers, which can be employed for an unambiguous differentiation. To date, the comparison of venom compositions from related species in the context of taxonomic classification is still a controversial debate [57]. In the following, we compare the venom composition of the two related Nose-Horned vipers, Vat and Vam, at the level of subspecies that may aid in recognizing of a close relationship, which could help to a potential taxonomic assessment.

The close phylogenetic relationship of the two studied snakes was previously implied by Ursenbacher et al. [39] using molecular phylogeography. Our study attempts to underpin the implied taxonomic status of Vat by comparative analysis at the venomic level, underscored by the comparison of the UV chromatograms (see Figure 3) as well as the TICs (see Figure 2) of the venoms. The curve progression and peak distribution in the chromatograms show a superimposable arrangement containing the same toxin families, which was confirmed by SDS-PAGE followed by similar de novo sequencing as described above (see Table 1 and Tables S1 and S2).

A closer look at the intact mass profiling exhibits the svMP-i pEKW and pENW as most abundant peptide part in both venoms, but, with ca. 7.0% and 1.0%, they are more prominent in Vam then in Vat (3.1% and 0.4%). On the other hand, the major peptide of fraction 11 (Vat *m*/*z* 3796.75 and Vam *m*/*z* 3796.73) with 0.9% to 0.4% is twice as abundant in Vat, as *m*/*z* 1143.64 (Vat) and *m*/*z* 1144.43 (Vam) as major Vat peptide in fractions 12, 13 and 14. In total, the snakes have eight close related peptide masses. This shows that the lower molecular masses are in the main contents similar, but differ strongly between the studied venoms in the lower abundant peptides and mostly in the abundance of 3–9 kDa masses. Additionally, the intact mass profiling exemplarily revealed six proteins from the venoms of Vat and Vam that are either in part or fully identical between these two vipers, as well as matched database entries for other species members (see Figure 7). Two molecular masses ~24 kDa were observed each in peak 19 of Vat (24,652.41 Da and 24,751.38 Da) and peak 21 of Vam (24,653.40 Da and 24,749.41 Da), which were both determined by de novo sequencing as CRISPs. The remaining four molecular masses ~13 kDa belong to the PLA_2_ family and were found in the strong peaks 17 and 20 of Vat (13,552.83 Da, 13,589.76 Da, 13,623.69 Da and 13,675.78 Da), and in peaks 19, 23 and 24 (13,552.82 Da, 13,589.75 Da, 13,623.69 Da and 13,675.81 Da) of Vam (Figures S6–S13), respectively.

The previously mentioned molecular mass of 13,623.69 Da identified from Vat (peak 20) and Vam (peak 23) was assigned to the closely related acidic phospholipase A_2_ homolog Vipoxin A chain (Uniprot-ID: P04084, including oxidized cysteines) M_av_ = 13,625.04 Da of the *V. a. meridionalis* [64]*.* Until now, Vipoxin was known as one of the most abundant components from the two other *Vipera ammodytes* subspecies [41]. In both tested venoms, these fractions were also the most abundant peaks, which underscore the importance of this toxin in the general venom composition. Additionally, by de novo sequencing, we identified fragments of the Vipoxin B chain in venoms of Vat (peak 18) and Vam (peak 20). The intact mass profiling further shows only for Vat (peak 18) a molecular mass of 13,813.21 Da that correlates to the average mass of the basic phospholipase A_2_ Vipoxin B chain (Uniprot-ID: P14420, including oxidized cysteines) M_av_ = 13,813.77 Da of the *V. a. meridionalis*. Furthermore, the measured molecular mass of 13,552.8 Da in both venoms mass-correlates with the neutral phospholipase A_2_ Ammodytin I2 (Uniprot-ID: P34180, including oxidized cysteines) M_av_ = 13,553.30 Da of *V. a. ammodytes* [65]. The presence of the same metalloprotease inhibitors and identical fragments of the precursor in both snake venoms, mentioned before, are further indicators of a close kinship.

Considering the study by Georgieva et al. [41] on the venom proteomes of *V. a. ammodytes* and *V. a. meridionalis*, we could compare our datasets in a wider context of the venoms of these subspecies. Georgieva et al. showed that several PLA_2_s constitute an important part of the venoms, which we also determined in high quantities, e.g., Vammin A, several Ammodytin variants and Vipoxin B [41]. In addition, from venoms of Vat and Vam, we could also identify several venom families shared between the other *V. ammodytes* subspecies members, such as PLA_2_, LAAO, growth factors as well as serine- and metalloproteinases. All these findings, which are solely based on the comparative venom analysis of four snakes of the same species, could be an indicator for a closer relationship and help to make a classification of the four snakes of *Vipera ammodytes* as subspecies comprehensible.

On the other hand, a view to other related *Vipera* species that are sharing the geographical habitat with the *Vipera ammodytes* species in parts show remarkable differences in the venom composition. For example, the wide distributed *Vipera berus berus*, which was recently compared to the *V. a. ammodytes*, exhibit svSP (31%) as the main toxin family followed by svMP (19%). In contrast, the most abundant families of the Vat and Vam play a subordinate role in the *V. b. berus* venom with 10% for PLA_2_ or were not even detected in the case of the VEGF-F [51]. Furthermore, the venom composition of the close related *Vipera* species *Vipera anatolica* is focused on the presence of the toxin families svMP (42%) and CRISP (16%) with a similar occurrence of PLA_2_ and VEGF-F to *V. b. berus* [14].

**Figure 4:**
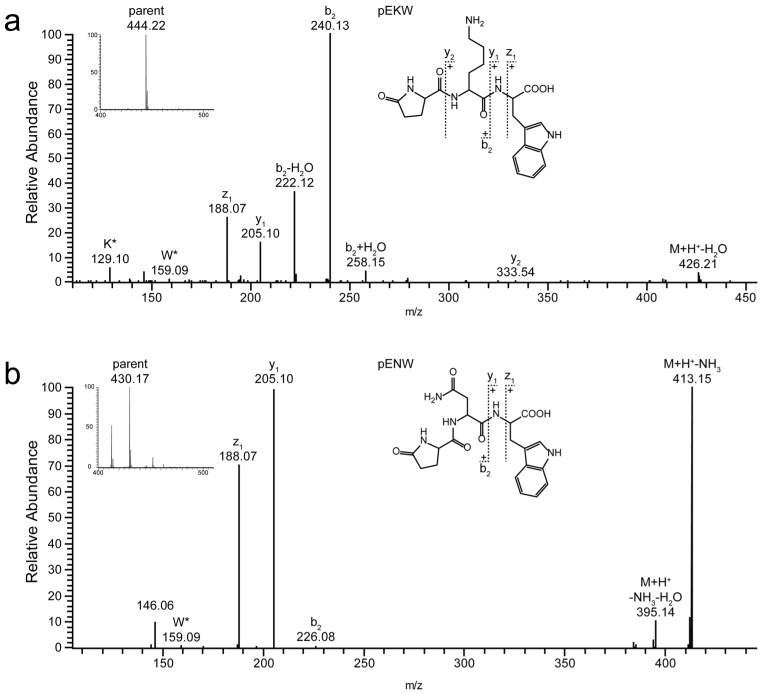
CID-MS/MS spectra of two snake venom metalloproteinase (svMP) inhibitor tripeptides. The two snake venom metalloproteinase inhibitor tripeptides: (**a**) pEKW; and (**b**) pENW were identified by intact mass profiling in the venoms of Vat and Vam (here Vat). The HPLC-ESI-MS1 spectra of selected parent ions are shown at the left corner. Standard fragmentation ions were indicated at the ion mass peaks and amino acid related ions by asterisked single letter code.

**Figure 5:**
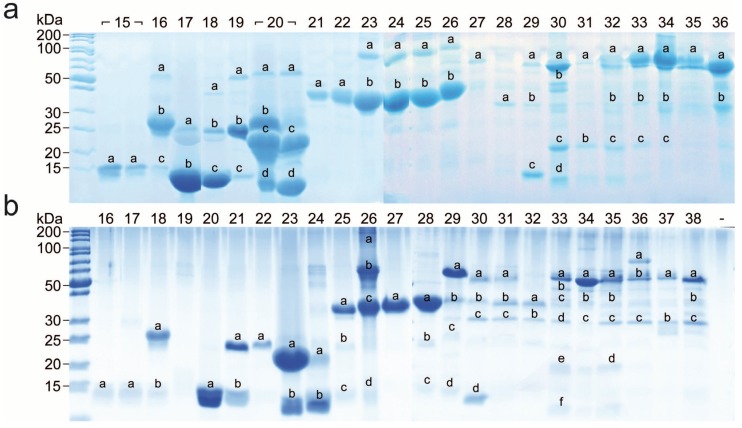
Venom fraction analysis of *V. a. transcaucasiana* and *V. a. montandoni* by SDS-PAGE. The RP-HPLC fractions (indicated above the lane are based on Figure 3) of the (**a**) Vat and (**b**) Vam venoms were analyzed by SDS-PAGE under reducing conditions. Alphabetically marked bands per line were excised for subsequent tryptic in-gel digestion.

**Figure 6:**
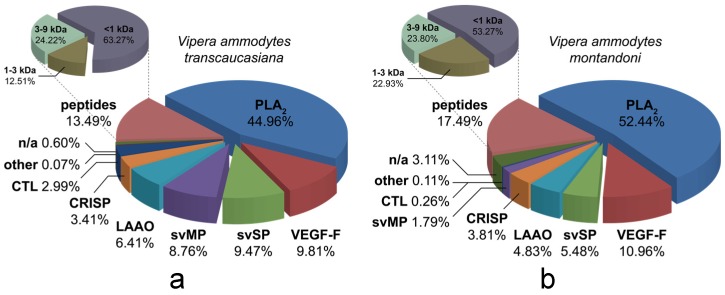
Semi-quantitative venom composition of *V. a. transcaucasiana* and *V. a. montandoni*. The relative occurrence of different toxin families of: (**a**) Vat and (**b**) Vam are represented by pie charts. Identification of phospholipases A2 (PLA_2_, blue), vascular endothelial growth factors (VEGF-F, red), snake venom serine proteases (svSP, green), snake venom metalloproteases (svMP, violet), l-amino acid oxidases (LAAO, light blue), cysteine rich secretory proteins (CRISP, orange), C-type lectin like proteins (CTL, dark blue), other proteins (other, dark red), unknown proteins (n/a, dark green) and peptides (light red). Groups of different peptide sizes are summarized in an additional pie chart as percentages of the total peptide content and clustered to <1 kDa (dull purple), 1–3 kDa (dull brown) and 3–9 kDa (dull green).

**Figure 7:**
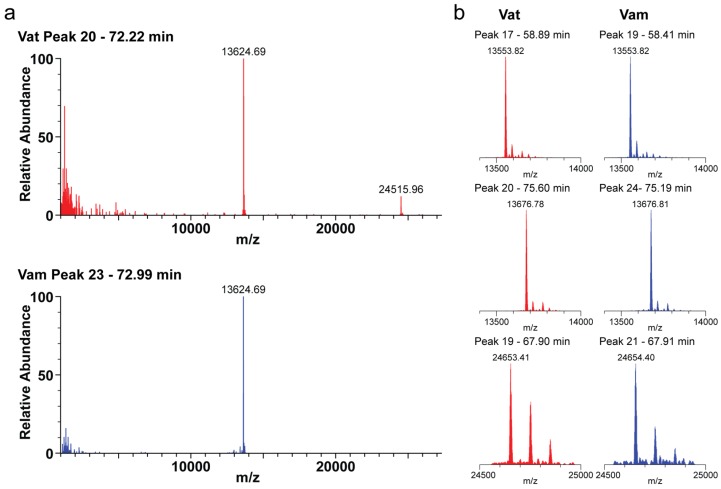
Comparative venom analysis of *V. a. transcaucasiana* and *V. a. montandoni*. Intact mass profiling of crude venoms from Vat (red) and Vam (blue) shows several identical masses [M + H]^+^ of different toxin families: (**a**) Vipoxin A chain in peak 20 (Vat) and peak 23 (Vam); and (**b**) from top to bottom: two phospholipase A_2_ (PLA_2_, ~13–14 kDa) in peaks 17 and 20 (Vat) and in peaks 19 and 24 (Vam), as well as one CRISP (~25 kDa) in peak 19 (Vat) and peak 21 (Vat).

Even if there are many similarities in the comparative analysis of the Vat and Vam venoms, some small differences remain, especially in the peptide content and in the protease pattern as well as in the LAAOs, which could be considered as parameters to distinguish between subspecies. However, venom compositions are susceptible to variation due to the influence of various factors (e.g., age, diet, sex and geographic origin) and could be more likely attributed to the occurrence of intraspecific variations [58,59,60,61,62]. The venom variations influenced by diet or habitat are not suspected in the cases of *V. a. transcaucasiana* and *V. a. montandoni*, whose analyzed specimens share the same geographical origin, but due to the small pooled sample size of each subspecific population, complete variation compensation cannot be excluded. Additionally, the Vat mass profile shows two dominant peaks (peak 17 and 18), which includes PLA_2_s (13,813.21 Da and 13,917.24 Da) that could not be detected in the Vam venom profile. In contrast, the Vam venom contains two abundant peaks (peaks 20 and 22) containing a PLA_2_ (13,889.25 Da) and a CRISP (24,546.04 Da) as a major difference that are missing in the Vat profile (see Figure 2 and Figure 4). These small differences, even between subspecies, play a crucial role in the development of effective antidotes and the understanding of reduced effects of polyvalent antivenoms. For example, a study of *V. a. ammodytes* antidotes has shown strong reduced neutralization potency against *V. a. montandoni* [44].

### 2.3. Cytotoxicity Screening

Snake venoms constitute complex mixtures of enzymes, peptides and proteins with a high toxicity potential, which can selectively and specifically act on various cellular targets by modulating the physiological function. This turns snake venoms into an attractive source for potential anticancer agents [66]. As part of our ongoing studies on Turkish snake venoms, the potency against various human cancer cells, and the cytotoxicities of the crude venom for *V. a. transcaucasiana* and *V. a. montandoni* were tested on a panel of cancer cell lines together with non-cancerous cell lines in a MTT assay. For *V. a. transcaucasiana* and *V. a. montandoni* crude venoms the MTT assay resulted as IC_50_ values of 1.34–22.75 µg/mL and 0.06–50.00 µg/mL (see Table 2), respectively.

**Figure 8:**
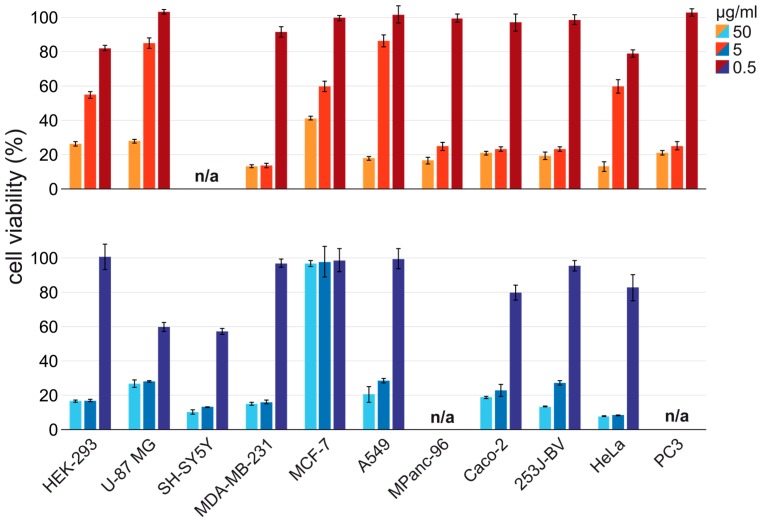
MTT human cell viability after 48 h crude venom treatment. Cytotoxicity of: *V. a. transcaucasiana* (red) (**top**); and *V. a. montandoni* (blue) (**bottom**) crude venom in three concentrations (50, 5 and 0.5 μg/mL) against different human cell lines. Cell viability was measured by MTT assay after 48 h at 570 nm. Noncancerous human cells: HEK-293 (human embryonic kidney). Cancerous human cells: U-87 MG (epithelial-like glioblastoma-astrocytoma); SH-SY5Y (neuroblastoma); MDA-MB-231 (breast epithelial adenocarcinoma); A549 (lung adenocarcinoma); MPanc-96 (pancreas adenocarcinoma); MCF-7 (epithelial breast adenocarcinoma); CaCo-2 (epithelial colorectal adenocarcinoma); 253-JBV (bladder carcinoma); HeLa (epithelial cervical carcinoma); PC-3 (prostate adenocarcinoma). Not tested cell lines by n/a and error in mean ± SD.

The crude venoms of both snakes show a similar activity against breast (MDA-MB-231), colon (Caco-2) and bladder (253J-BV) cancer cell lines. *V. a. montandoni* shows a high cytotoxicity against four cell lines (HEK-293, U-87 MG, A549 and HeLa) out of the eight tested (see Figure 8). The determination of the IC_50_ shows strong differences of the closely related vipers against the MCF-7 breast cancer cells and the A549 lung cancer cells. The *V. a. transcaucasiana* venom was found to have an IC_50_ of 21.75 ± 2.45 µg/mL and 18.03 ± 2.09 µg/mL against MCF-7 and A549 cells, respectively. In contrast, the *V. a. montandoni* venom has an IC_50_ >50.00 µg/mL and 4.40 ± 0.03 µg/mL against MCF-7 and A549 cells, respectively (see Table 3). Interestingly, the *V. a. transcaucasiana* crude venom exhibited the highest cytotoxic effect (1.84 ± 0.76 µg/mL) against the receptor triple negative breast cancer cells MDA-MB-231 in comparison to *V. a. montandoni* and the 2.6-fold less active positive control (4.80 ± 1.10 µg/mL), while both venoms are less toxic to the second tested breast cancer cells MCF-7 (ER negative, PR positive, HER2 positive) with IC_50_ values of 21.75 ± 2.45 µg/mL and >50.00 µg/mL to Parthenolide (6.01 ± 1.15 µg/mL). The subtypes of breast cancer have been generally identified based on the presence of three receptors: estrogen receptor (ER), progesterone receptor (PR) and human epidermal growth factor receptor-2 (HER-2) [67].

**Figure 9:**
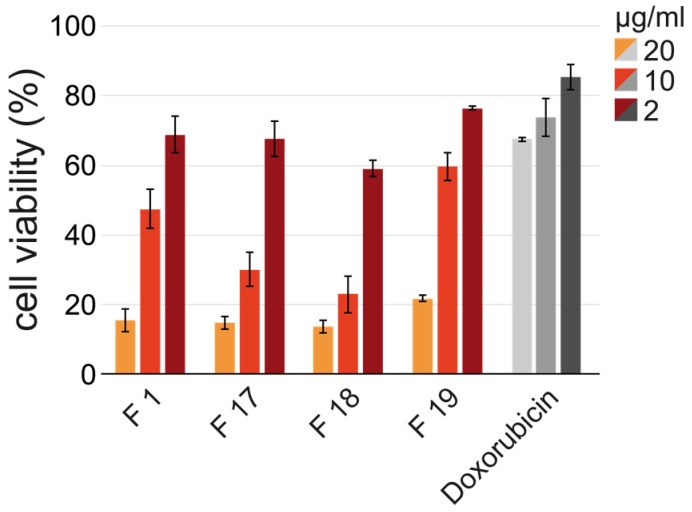
MTT human cell viability after 48 h *V. a. transcaucasiana* venom fraction treatment. Cytotoxicity of selected *V. a. transcaucasiana* venom HPLC fractions (F1,17–19) in three concentrations (20 μg/mL, 10 μg/mL and 2 μg/mL) against MDA-MB-231 breast adenocarcinoma epithelial cell line. Cell viability was measured by MTT assay after 48 h at 570 nm. Doxorubicin was used as reference compound and error in mean ± SD.

Based on these markers, a classification of breast cancer tumors as hormone receptor positive, HER-2/Neu amplified tumors, and those which do not express ER, PR and do not have a HER-2/Neu amplification were defined as triple-negative breast cancer (TNBC). This might have a significant role for patient-tailored treatment strategies, as TNBC demonstrates approximately 10–15% of all breast cancers and patients with TNBC have an unsuccessful outcome compared to the other breast cancer subtypes [68]. Unfortunately, the tested crude venoms also exhibit a comparable cytotoxic effect against the tested non-cancerous kidney cells with values of 1.34 ± 0.72 and 3.55 ± 0.61 µg/mL. The comparison of further non-cancerous to attributed cancerous cell lines is part of ongoing due to restricted sample size. Nevertheless, we suspect that the high toxic effect on non-cancerous cell lines can be overcome by developing a targeted drug delivery system for potential treatment followed by detailed studies for each drug candidate. According to the MTT assay, *V. a. transcaucasiana* venom showed active venom fractions against the human breast adenocarcinoma epithelial cells (MDA-MB-231) (see Figure S14). Fractions 1, 17, 18 and 19 exhibited the strongest cytotoxic effect on the triple negative MDA-MB-231 breast cancer cells with IC_50_ of 2.96 µg/mL to 9.22 µg/mL (see Figure 9 and Table 3).

Mass spectrometric analysis of the bioactive fractions identified fraction 17 as an Ammodytin I2(A) variant, fraction 18 as a Vipoxin chain B or Vaspin basic subunit variant and fraction 19 as a cysteine-rich venom protein. Fraction 1 is a mixture of various small peptides with still unknown sequence. The effectivity and specificity of snake venom phospholipases against cancer cells are known [69]. The cytotoxicity of the CRISP in fraction 19 is due to the blocking ability against several ion channels [70,71].

## 3. Conclusions

Here, we report on the first proteomic characterization of the venoms from *Vipera ammodytes transcaucasiana* and *Vipera ammodytes montandoni* by using a combined mass spectrometry-guided approach. The initial intact mass profiling of the venoms facilitated the detection of ~50 venom components for *V. a. transcaucasiana* and ~59 venom components for *V. a. montandoni*. Additionally, the intact mass profiling revealed the presence of two tripeptide metalloprotease inhibitors and their precursors in the venoms, which would not have been detected by the bottom-up approach. However, due to the limited applicability to high molecular mass compounds the analysis was further expanded to the bottom-up approach. The de novo sequencing showed for both snake venoms the presence of 11 major *Viperidae* toxin families with the exception of Kunitz type proteinase inhibitors and hyaluronidases, which are not present in either venom.

The comparative analysis by initial mass profiling in combination with the well-established bottom-up protocol revealed strong similarities in the venom composition of the two studied snake venoms of *V. a. transcaucasiana* and *V. a. montadoni*. While related toxin families or even identical proteins could be identified proving a close relationship of the proteome and under consideration of intraspecific venom variation, small differences in the venom compositions in turn could be parameters for a differentiation into subspecies.

In summary, the mass spectrometry-guided comparative analysis of the venom proteome extended by intact mass profiles provides an excellent method to highlight close relationships of venomous snakes at the proteome level and especially in combination with top-down venomics this would give you an even more detailed picture of venom diversity. Hence, the investigation of venoms for the phylogenetic analysis, using a combination of chromatographic, electrophoretic and different mass spectrometric techniques, is very sensitive and fast. In this context, reliable databases are an important basis for the de novo identification and the variation of the venom composition by differences in age and food supply should always be considered as a critical point [72]. The future transcriptomic analysis of the venom glands represents a valuable technique, which could support the proteomic analysis and elucidate the venom proteome in its entirety. The complete analysis of the venom proteome of *Vipera ammodytes transcaucasiana* in connection with mitochondrial DNA could finally clarify the controversial question about the taxonomic status.

Finally, the preliminary in vitro cytotoxicity screening against various cancer cell lines for the crude venoms and components for *V. a. transcaucasiana* demonstrated significant cytotoxic effects on the triple negative MDA MB 231 breast cancer cells with IC_50_ of 2.96 µg/mL to 9.22 µg/mL for an Ammodytin I2(A) variant, a Vipoxin chain B or Vaspin basic subunit variant and a cysteine-rich venom protein. Nevertheless, the high toxic effect also against non-cancerous cell lines requires the development of a targeted drug delivery system for potential treatment.

## 4. Materials and Methods

### 4.1. Collection and Preparation of Venom Samples

Venom samples of the *V. a. transcaucasiana* were collected in June 2015 from four specimens in total, one from Işık Mountain (Çerkeş district, Çankırı Province, Turkey) and three from Çavuş Mountain (Sivas Province, Turkey). *Vipera ammodytes montandoni* venom samples were collected from one individual in the Tekirdağ province and one in the Kırklareli province (Turkish Thrace) in April 2016. Crude venoms were extracted, using a paraffin-covered laboratory beaker without exerting pressure on the venom glands, pooled for each subspecies and lyophilized. Ethical permission (Ege University, Animal Experiments Ethics Committee, 2010#43) and special permission (2011#7110) for field studies from the Republic of Turkey, Ministry of Forestry and Water Affairs were received.

### 4.2. Determination of Protein Content

Protein concentrations were determined from diluted venom sample (1 or 2 mg/mL) in ultrapure water by Micro-BCA (Bicinchoninic Acid) Protein Assay using a UV/Vis spectrophotometer (Thermo-Scientific, Darmstadt, Germany) at a wavelength of λ = 595 nm. Bovine serum albumin (BSA) was used as a reference.

### 4.3. Cell Culture and In Vitro Cytotoxicity Assay

The following human cell lines were used for determination of cytotoxicity: noncancerous cells: HEK293 (human embryonic kidney); cancerous human cells: U87MG (epithelial-like glioblastoma-astrocytoma); SHSY5Y (neuroblastoma); MDA-MB-231 (breast epithelial adenocarcinoma); A549 (lung adenocarcinoma); MPanc-96 (pancreas adenocarcinoma); MCF-7 (epithelial breast adenocarcinoma); CaCo-2 (epithelial colorectal adenocarcinoma); 253-JBV (bladder carcinoma); HeLa (epithelial cervical carcinoma); PC-3 (prostate adenocarcinoma). All cell lines were purchased from the US American Type Culture Collection (ATCC, Manassas, VA, USA) except for 253J-BV cells, which were obtained from Creative Bioarray (Shirley, NY, USA). All cells were cultivated in Dulbecco’s modified Eagle’s medium F12 (DMEM/F12), supplemented with 10% fetal bovine serum (FBS), 2 mM/L glutamine, 100 U/mL of penicillin and 100 mg/mL of streptomycin (Gibco, Visp, Switzerland). The in vitro cytotoxicity testing with crude venoms and fractions was performed according to the protocol of Nalbantsoy and Hempel et al. [15]. The morphological changes of the cells after treatment with crude venom or its fractions were observed with an inverted microscope (Olympus, Tokyo, Japan) compared to the control group following for 48 h.

### 4.4. Determination of Half Maximal Inhibitory Concentration (IC_50_)

The IC_50_ values were calculated by fitting the data to a sigmoidal curve and using a four-parameter logistic model and presented as an average of three independent measurements. The IC_50_ values were reported at 95% confidence interval, and calculations were performed using Prism 5 software (GraphPad5, San Diego, CA, USA). The values of the blank wells were subtracted from each well of treated and control cells and half maximal inhibition of growth (IC_50_) were calculated in comparison to the untreated controls.

### 4.5. Preparation of Venom Samples for Intact Mass Profiling

The crude venoms were dissolved in aqueous 1% (*v*/*v*) formic acid (HFo) to a final concentration of 10 mg/mL, and centrifuged at 20,000× *g* for 5 min to spin down insoluble content. Dissolved venoms were then mixed with 30 µL of citrate buffer (0.1 M, pH 4.0). The samples were mixed with an equal volume of 1% aqueous formic acid and centrifuged at 20,000× *g* for 5 min. Subsequently, samples were submitted to HPLC-high-resolution (HR) ESI-MS/MS measurements.

### 4.6. Intact Mass Profiling

The intact mass profiling was performed by LC-ESI-HR-MS experiments on an LTQ Orbitrap XL mass spectrometer (Thermo, Bremen, Germany) coupled to an Agilent 1260 HPLC system (Agilent, Waldbronn, Germany) using a Supelco Discovery 300 Å C18 (2 × 150 mm, 3 µm particle size) column. The instrument settings for the HPLC system and ESI-MS were adopted from Nalbantsoy and Hempel et al. [15]. The intact mass profiles were inspected with the Xcalibur Qual Browser (Thermo Xcalibur 2.2 SP1.48, Thermo Fisher Scientific, Waltham, MA, USA) and deconvolution of isotopically resolved spectra was carried out by using the XTRACT algorithm of Xcalibur Qual Browser. The protein assignment was done by comparison to the retention time of the HPLC run and corresponding LC-MS/MS information from SDS-PAGE trypsin digests.

### 4.7. Bottom-Up Venomics

The lyophilized crude venoms (4 mg) were dissolved to a final concentration of 20 mg/mL in aqueous 3% (*v*/*v*) ACN with 1% (*v*/*v*) HFo and centrifuged at 20,000× *g* for 5 min to spin down insoluble content. The supernatant was loaded onto a semi-preparative reversed-phase HPLC with a Supelco Discovery BIO wide Pore C18-3 column (4.6 × 150 mm, 3 µm particle size) using an Agilent 1260 Low Pressure Gradient System (Agilent, Waldbronn, Germany). The column was operated with a flow rate of 1 mL/min and performed using ultrapure water with 0.1% (*v*/*v*) HFo (buffer A) and ACN with 0.1% (*v*/*v*) HFo (buffer B). The technical settings and bottom-up workflow was performed according to the protocol of Nalbantsoy and Hempel et al. [15] with the following amendments. After the chromatographic separation of the crude venoms, the vacuum-dried peak fractions were submitted to a SDS-PAGE with a content of 15% polyacrylamide. Afterwards the coomassie-stained band were excised and reduced, via in-gel trypsin digestion, with freshly prepared dithiothreitol solution (100 mM DTT in 100 mM ammonium hydrogencarbonate, pH 8.3, heated for 30 min at 56 °C) and alkylated with iodoacetamide (55 mM IAC in 100 mM ammonium hydrogencarbonate, pH 8.3, stored for 30 min at 25 °C in the dark). The peptides were extracted with 100 µL aqueous 30% (*v*/*v*) ACN just as 5% (*v*/*v*) HFo for 15 min at 37 °C. The supernatant was vacuum dried (Thermo speedvac, Bremen, Germany), re-dissolved in 20 µL aqueous 3% (*v*/*v*) ACN with 1% (*v*/*v*) HFo and submitted to HPLC-MS/MS analysis.

The bottom-up analysis was performed with an Orbitrap XL mass spectrometer (Thermo, Bremen, Germany) via an Agilent 1260 HPLC system (Agilent Technologies, Waldbronn, Germany) using a reversed-phase Grace Vydac 218MSC18 (2.1 × 150 mm, 5 µm particle size) column. The pre-chromatographic separation was performed with the following settings: After an isocratic equilibration (5% B) for 1 min, the peptides were eluted with a linear gradient of 5–40% B for 10 min, 40–99% B for 3 min, washed with 99% B for 3 min and re-equilibrated in 5% B for 3 min.

LC-MS/MS data files (.raw) were converted to mgf-files using MSConvert GUI of the ProteoWizard Software Foundation (ProteoWizard package, version 3.0.10577, Los Angeles, CA, USA) and annotated by DeNovo GUI [73] (ProteoWizard package, version 1.15.8, Los Angeles, CA, USA) with the following settings: fixed modifications: carbamidomethyl Cys (+57.02 Da); variable modifications: acetylation of Lys (+42.01 Da) and phosphorylation of Ser and Thr (+79.97 Da). The deduced amino acid sequences were squared against a non-redundant protein NCBI database of *Viperidae* (taxid:8689) using BLASTP [74] (http://blast.ncbi.nlm.nih.gov).

### 4.8. Data Accessibility 

Mass spectrometry proteomics data (.mgf, .raw and output files) have been deposited with the ProteomeXchange Consortium [75] (http://proteomecentral.proteomexchange.org) via the MassIVE partner repository under Project Name “Venomics of the *V. a. transcaucasiana* and *V. a. montandoni*” and data set identifier PXD007609.

### 4.9. Relative Toxin Quantification

The quantification of venom composition is based on the RP-HPLC peak integration (UV_214nm_) in comparison to the total integral of all analyzed peaks based on the protocol of Juarez et al. [76]. In the case of HPLC co-eluting toxins components, the SDS-PAGE band ratio of optical intensities and densities was, respectively, used for emphasis of peak integral portion [17,22,23].

## Figures and Tables

**Table 1:** Venom proteins and peptides identified from *Vipera ammodytes transcaucasiana* and *Vipera ammodytes montandoni.* Venomic components were assigned by crude venom intact mass profiling and bottom-up approach. Peak numbers and retention time (RT) based on RP-HPLC (see Figure 3) and TIC (see Figure 2) annotation. SDS-PAGE and intact mass profile analysis provided the molecular weight. Most abundant masses are asterisked (*). For peptide fractions, only the most abundant masses were noted. Detailed lists of all exhibit masses are mentioned in Tables S1 and S2.

*V. a. transcaucasiana*					*V. a. montandoni*				
RT (min)	Fraction No.	Protein Species	SDS PAGE M_av_ (kDa)	Most Abundant IMP (*m*/*z*)	RT (min)	Fraction No.	Protein Species	SDS PAGE M_av_ (kDa)	Most Abundant IMP (*m*/*z*)
2.14	1	unknown	-	571.25 *	-	-	-	-	-
3.45	2	unknown	-	484.20 *	3.46	1	unknown	-	484.21 *
-	-	-	-	-	6.45	2	svMP-i, unknown	-	1234.65 *
12.86	3	unknown	-	600.32 *	-	-	-	-	-
15.48	4	svMP-i, svMP unknown	-	547.22 *	15.43	3	svMP-i, unknown	-	444.22 *
19.47	5	svMP, unknown	-	814.35 *	19.19	4	svMP-i, unknown	-	444.23 *
21.08	6	svMP-i, unknown	-	444.22 *	20.48	5	svMP-i, unknown	-	444.22 *
24.49	7	svMP-i, unknown	-	430.17 *	24.52	6	svMP-i, unknown	-	430.17 *
27.85	8	unknown	-	569.28 *	26.99	7	unknown	-	1072.60 *
-	-	-	-	-	31.95	8	svMP-i, unknown	-	3930.96 *
33.07	9	svMP-i, unknown	-	5775.64 *	33.02	9	unknown	-	3761.72 *
33.86	10	unknown	-	4176.85 *	33.91	10	unknown	-	3796.72 *
34.95	11	BPP, unknown	-	3769.75 *	35.20	11	unknown	-	3796.73 *
36.97	12	unknown	-	1143.64 *	36.44	12	unknown	-	1144.62 *
-	-	-	-	-	39.50	13	unknown	-	1314.73 *
40.43	13	unknown	-	1143.64 *	40.75	14	unknown	-	1144.62 *
42.44	14	svMP-i, unknown	-	1143.64 *	42.94	15	unknown	-	-
46.90	15	VEGF-F	15 *	-	47.16	16	VEGF-F	14 *	1159.59 *
-	-	-	-	-	48.69	17	VEGF-F	14 *	1159.59 *
54.79	16	VEGF-F, svMP, unknown	15, 27 *, 50	10,676.97, 21,311.88 *	55.03	18	unknown	14, 25 *	21,199.58 *, 21,298.85
-	-	-	-	-	58.69	19	unknown	-	13,553.82 *, 13,590.75
61.54	17	PLA_2_, unknown	13 *, 25, 40	13,553.83 *, 13,590.76, 13,814.21, 13,842.19, 13,911.15	62.75	20	PLA_2_	13 *	13,553.82 *, 13,890.28, 13,988.22
63.85	18	PLA_2_	13 *, 25, 40	13,918.28 *, 14,016.22	-	-	-	-	-
66.42	19	PLA_2_, CRISP	13, 25 *, 50	12,346.55, 24,653.41 *, 24,752.38, 24,848.30	66.68	21	PLA_2_, CRISP	13.23 *	24,654.40 *, 24,750.41
-	-	-	-		69.08	22	CRISP	23 *	24,547.04 *
71.28	20	PLA_2_, svMP, CRISP	13, 22.5 *, 27.5, 60	13,624.69, 13,625.73, 13,676.78, 24,515.96 *	71.38	23	PLA_2_	13, 21 *	13,624.69
-	-	-	-	-	74.21	24	PLA_2_	13 *, 21	13,676.81 *
-	-	-	-	-	76.93	25	PLA_2_, svSP, unknown	15, 23, 37 *	-
78.24	21	svSP	35 *	-	-	-	-	-	-
78.90	22	unknown	35 *	-	-	-	-	-	-
79.43	23	svSP	35 *, 85	-	79.74	26	svSP, unknown	15, 37 *, 60, >200	32,026.88 *, 32,899.08
80.23	24	svSP	35 *, 85	-	80.39	27	svSP	37 *	32,686.16 *, 35,124.93
80.95	25	svSP	35 *, 85	-	81.17	28	CRISP, svSP, unknown	15, 25, 37 *	32,686.34 *, 33,342.02
81.59	26	svSP	40 *, 100	-	-	-	-	-	-
84.01	27	svMP	70 *	-	-	-	-	-	-
85.34	28	svSP	35 *	-	84.65	29	svMP, trypsin-like, unknown	15, 25, 37, 60 *	24,547.96, 27,654.98
87.17	29	CTL, svSP, PDE	13 *, 35, 65	16,108.34 *, 16,208.30	87.19	30	CTL, svSP, svMP	13 *, 30, 37, 55	13,890.25 *
-	-	-	-	-	88.14	31	svSP, unknown	30, 37 *, 55	-
-	-	-	-	-	89.12	32	svSP, unknown	30 *, 37	-
92.85	30	CTL, LAAO	13, 20, 40, 60 *	-	93.23	33	CTL, svSP, LAAO	11, 20, 30, 37, 50, 55 *	-
93.88	31	CTL, LAAO	20, 60 *	-	94.64	34	LAAO	30, 37, 55 *	-
101.76	32	CTL, DI, svMP, unknown	20, 35, 60 *	-	-	-	-	-	-
103.42	33	svSP, svMP, unknown	20, 35, 60 *	-	103.23	35	CTL, svSP, LAAO	11, 20, 30, 37, 50 *	-
104.82	34	svMP, LAAO, svSP	20, 35, 65 *	-	104.83	36	aminopeptidase, svMP, LAAO, unknown	30, 50 *, 70	-
105.70	35	svMP	65 *	-	106.47	37	svMP, LAAO	30, 50 *	-
108.84	36	svSP, LAAO, svMP	35, 60 *	-	108.82	38	unknown	30, 37, 50 *	-

**Table 2:** IC_50_ values of *Vipera ammodytes transcaucasiana* and *Vipera ammodytes montandoni* venoms against various human cell lines. The half maximal inhibitory concentration (IC_50_ in µg/mL) for the venom of Vat and Vam were determined after 48 h exposure. Parthenolide was used as reference compound. Noncancerous human cells: HEK293 (human embryonic kidney). Cancerous human cells: U87MG (epithelial-like glioblastoma-astrocytoma); SHSY5Y (neuroblastoma); MDA-MB-231 (breast epithelial adenocarcinoma); A549 (lung adenocarcinoma); MPanc-96 (pancreas adenocarcinoma); MCF-7 (epithelial breast adenocarcinoma); CaCo-2 (epithelial colorectal adenocarcinoma); 253-JBV (bladder carcinoma); HeLa (epithelial cervical carcinoma); PC-3 (prostate adenocarcinoma). A minus (−) mentioned not tested cell lines and error in mean ± SD.

Cell Line	*V. ammodytes transcaucasiana* IC_50_ (µg/mL)	*V. ammodytes montandoni* IC_50_ (µg/mL)	Parthenolide IC_50_ (µg/mL)
HEK293	1.34 ± 0.72	3.55 ± 0.61	1.23 ± 0.24
U87MG	6.02 ± 1.38	1.02 ± 0.20	3.33 ± 0.59
SHSY5Y	-	0.06 ± 0.01	0.15 ± 0.01
MDA-MB-231	1.84 ± 0.76	2.36 ± 0.20	4.80 ± 1.10
MCF-7	21.75 ± 2.45	>50.00	6.01 ± 1.15
A549	18.03 ± 2.09	4.40 ± 0.03	4.42 ± 0.87
MPanc-96	22.75 ± 2.25	-	4.70 ± 0.87
CaCo-2	4.21 ± 0.96	1.82 ± 0.14	4.90 ± 1.10
253J-BV	4.22 ± 1.41	3.00 ± 1.98	5.45 ± 1.16
HeLa	6.14 ± 1.12	1.27 ± 0.20	5.75 ± 1.07
PC3	6.95 ± 1.19	-	3.33 ± 0.96

**Table 3:** IC_50_ values of selected *V. a. transcaucasiana* HPLC fractions against MDA-MB-231 cells. The half maximal inhibitory concentration (IC_50_ in µg/mL) for the HPLC fractions 1, 17, 18 and 19 of Vat venom against MDA-MB-231 breast adenocarcinoma epithelial cell line. Doxorubicin was used as reference compound and error in mean ± SD.

Samples ID	*V. ammodytes transcaucasiana* Fraction				Doxorubicin
	1	17	18	19	
**IC_50_ (µg/mL)**	5.92 ± 0.14	3.98 ± 0.85	2.96 ± 0.38	9.22 ± 0.62	>20.00

## Supplementary Materials

The following are available online at www.mdpi.com/2072-6651/10/1/23/s1, Figures S1–S5: Tripeptide precursor fragments of *V. a. montandoni* and *V. a. transcaucasiana*, Figures S6–S9: Intact masses of *V. a. transcaucasiana*, Figures S10–S13: Intact masses of *V. a. montandoni*, Figures S14: Cytotoxicity analysis of fractionated components from *Vipera a. transcaucasiana*, Table S1: Venom proteins and peptides identified from *Vipera ammodytes transcaucasiana*, Table S2: Venom proteins and peptides identified from *Vipera ammodytes montandoni*.
