# Supplementary material for: Comparative Venomics of the Vipera ammodytes transcaucasiana and Vipera ammodytes montandoni from Turkey Provides Insights into Kinship
Source: Toxins (Basel). 2018 Jan 1;10(1):23. doi: 10.3390/toxins10010023 (PMC5793110; doi:10.3390/toxins10010023)
Supplement: Supplementary file 1 [file toxins-10-00023-s001.pdf]

Supplementary Materials: Comparative Venomics of the *Vipera ammodytes transcaucasiana* and *Vipera ammodytes montandoni* from Turkey Provides Insights into Kinship

Benjamin-Florian Hempel, Maik Damm, Bayram Göçmen, Mert Karis, Mehmet Anıl Oguz, Ayse Nalbantsoy and Roderich D. Süssmuth

Table S1. Venom proteins and peptides identified from *Vipera ammodytes transcaucasiana*. Assignment of venomic components by crude venom intact mass profiling (method A) and bottom-up (method C). RP-HPLC fractions with low molecular masses were additionally analyzed by intact mass profiling (method B). Peak numbers based on RP-HPLC (see Figure 4A) and TIC (see Figure 2A) annotation. Sequence tags were obtained de novo from MS/MS spectra and identified against a non-redundant *Viperidae* protein database (taxid: 8689) by BLASTP. SDS-PAGE and intact mass profile analysis provided the molecular weight.

Peak number	Method	SDS PAGE M_{av} [kDa]	Identification sequence tag	Protein species	Protein ID	BLAST E-value	NCBI accession number	intact mass m/z native avarage
**1**	A; B	-	-	-	unknown (peptide)	-	-	409.05, 442.12, 519.19, 549.21, 571.25, 611.20, 689.34, 1005.65, 1607.08
**2**	A; B	-	DEPLKK	-	unknown (peptide)	-	-	413.26, 484.20, 612.26, 967.40
**3**	A; B	-	-	-	unknown (peptide)	-	-	413.19, 485.70, 600.32, 617.80, 823.40, 1106.51, 1607.03
**4**	A; B	-	EDETPKM	svMP	metalloproteinase type III 6a	6.0E-04	JAS05414.1	413.36, 433.18, 490.20, 547.22, 607.27, 685.32, 773.49, 837.50, 849.36, 6424.04, 6693.16, 6793.24
		-	pEKW	svMP-i	tripeptide metalloproteinase inhibitor	-	-	
		-	GGGGGGW	svMP-i	endogenous tripeptide metalloproteinase inhibitor precursor	4.0E-01	AMB36336.1	
**5**	A; B	-	SEDYSETHYSPDWR	svMP	H3 metalloproteinase precursor 1	3.0E-07	AGL45259.1	
		-	PVSGNEL	svMP	metalloproteinase of class P-II MPII-3	2.8E-01	AMB36351.1	
		-	-	-	unknown (peptide)	-	-	457.11, 715.36, 814.35, 846.35

**6**	A; B	-	pEKW	svMP-i	svMP-i tripeptide	-	-	444.22, 448.22, 456.22, 460.22, 476.21, 486.23
**7**	A; B	-	pENW	svMP-i	svMP-i tripeptide	-	-	413.14, 430.17, 452.15, 706.38, 859.33, 1143.79, 3835.92, 4014.86, 4052.80, 4081.80
**8**	A; B	-	-	-	unknown (peptide)	-	-	569.28, 851.46, 907.44, 3035.36, 3943.82, 4014.88, 7486.42
**9**	A; B	-	PEGPPLMEPHE	svMP-i	endogenous tripeptide metalloproteinase inhibitor precursor	9.0E-07	AMB36336.1	569.28, 1232.56, 3859.92, 3931.00, 4296.04, 5775.64, 7470.42
**10**	A; B	-	-	-	unknown (peptide)	-	-	416.14, 723.35, 869.40, 3761.75, 3931.00, 4176.85, 8352.70
**11**	A; B	-	PPRCPGPKVPP	BPP	Bradykinin-potentiating peptide 12e	1.1E-01	P0DL03.1	723.36, 1143.64, 3380.75, 3796.75, 4861.17, 7470.46
**12**	A; B	-	-	-	unknown (peptide)	-	-	904.51, 932.51, 1143.64, 6970.90, 7488.48
**13**	A; B	-	-	-	unknown (peptide)	-	-	904.51, 932.51, 1143.64, 1472.76, 6785.75, 8351.72
**14**	A; B	-	QMPPPGPKVPPLK	svMP-i	endogenous tripeptide metalloproteinase inhibitor precursor	2.1E-02	AMB36336.1	904.51, 932.51, 1143.64, 7332.38, 14427.10
			PPQMPGPKVPP	svMP-i	endogenous tripeptide metalloproteinase inhibitor precursor	8.3E-02	AMB36335.1	
**15**	C	15	CSGCCTDESLK	VEGF-F	Vammin	3.0E-06	P67863.2	
			PFLEVHER			2.0E-03		
			366.21-PFLEVHER			3.0E-03		
			803.34-TVDLQIMR			3.0E-03		
**16a**	C	50	-	-	unknown (protein)	-	-	
**16b**	A; C	27	DASYFYCR	svMP	metalloproteinase	1.1E-01	ADI47645.1	21311.88, 21213.90

			GESYFYCR			1.0E-03		
**16c**	C	15	LLPNTLQFLLK	-	rho GTPase-activating protein 29	9.0E-03	XP_015684754.1	10676.97
			366.21-PFLEVHER	VEGF-F	Vammin	3.0E-03	P67863.2	
**17a**	C	40	-	-	unknown (protein)	-	-	
**17b**	C	25	NLYQFGNMIFK	PLA_2	Ammodytin I2(A) variant	4.0E-07	CAE47197.1	
			384.24-SYSNYGCYFDS-296.29		Vaspin basic subunit variant	3.0E-03	CAE47300.1	
			LAIYSYSFK			6.0E-04		
			NLFQFAK			6.4E-02		
**17c**	A; C	13	GTLLSYSNYGCYCGWGGK	PLA_2	Ammodytin I2(A) variant	4.0E-12	CAE47197.1	13553.83, 13590.76, 13814.21, 13842.19, 13911.15
			LGAICFGENLNTYDKK			2.0E-09		
			354.23-CFGENLNTYDKK			1.0E-07		
			NLYQFGNMIFK			4.0E-07		
			227.13-YQFGNFLFK			2.0E-01		
**18a**	C	40	LAIYYYSFK	PLA_2	Vipoxin Chain B	2.0E-04	1AOK_B	
			NLFQFAK		Vaspin basic subunit variant	6.4E-02	CAE47300.1	
**18b**	C	25	DICDCERVAANCLNGCLP	PLA_2	Vaspin basic subunit variant	1.0E-05	CAE47300.1	
			LGANCFHQNK			3.0E-03		
			LAIYYYSFK		Vipoxin Chain B	2.0E-04	1AOK_B	
**18c**	A; C	13	NLYQFGNMIFK	PLA_2	Ammodytin I2(A) variant	4.0E-07	CAE47197.1	13918.28, 14016.22
			VAANCFHQNK		Vaspin basic subunit variant	3.0E-05	CAE47300.1	
			LAIYYYSFK		Vipoxin Chain B	2.0E-04	1AOK_B	
**19a**	C	50	310.13-YVCQYCPAGNLIGK	CRISP	cysteine-rich venom protein	6.0E-09	XP_015678374.1	
			SVDFDSESPR			2.0E-05	P86537.1	
			LFWYPEAAA-470.22			3.4E-02		
**19b**	A; C	25	310.13-YVCQYCPAGNLIGK	CRISP	cysteine-rich venom protein	6.0E-09	XP_015678374.1	24653.41, 24752.38, 24848.3
			276.08-WYPEAAANVTR			3.0E-03		

**19c**	A; C	13	LAIYSYSFK	PLA_2	Vaspin basic subunit variant	6.0E-04	CAE47300.1	12346.55
**20a**	C	60	NLFQFGDMILQK	PLA_2	phospholipase A2 I acidic chain	9.0E-08	A60512	
			227.13-FQFGDFILQK		Vaspin acidic subunit (1) variant	2.0E-03	CAE47105.1	
			1110.6-TAIDFNGLITGK	svMP	group III metalloproteinase	2.0E-03	ADI47577.1	
			PCLTLYQCR			9.1E-02	ADW54342.1	
			786.47-PLVGVELWR			3.1E-01		
**20b**	C	27.5	ALYGCYCGWGGQGR	PLA_2	Vaspin acidic subunit (1) variant	7.0E-09	A60512	
			NLFQFGDMILQK			9.0E-08		
			326.20-SSLGENVNTYDK			1.0E-04	CAE47105.1	
			SVDFDSESPR	CRISP	cysteine-rich venom protein	2.0E-05	P86537.1	
			568.33-SSGENLYMSTSPMK			1.0E-05	B7FDI0.1	
			KPELQQDLLDLH-470.26			7.2E-01		
**20c**	A; C	22,5	NLFQFGDMILQK	PLA_2	Vaspin acidic subunit (1) variant	9.0E-08	A4VBF0.1	24515.96
			326.20-SSLGENVNTYDK			9.0E-05		
**20d**	A; C	13	HLSQFGDMINKK	PLA_2	Ammodytin I2(A) variant	2.0E-07	CAE47141.1	13624.69, 13625.73, 13676.78
			354.24-CFGENMNTYDQK			8.0E-07		
			KLLCFGENMNTYDQK			4.0E-06		
**21**	C	35	TLCAGLLQGGLDSCK	svSP	serine proteinase-like protein 2	2.0E-06	Q9PT40.1	
**22**	C	35	-	-	unknown (protein)	-	-	
**23a**	C	85	LMGWGTITTTK	svSP	enzymatically inactive serine proteinase-like protein SPH-1	3.0E-05	AMB36342.1	
			VVCAGIWQGGK		Nikobin	5.0E-06	E5AJX2.1	
**23b**	C	35	LMGWGTITTTK	svSP	enzymatically inactive serine proteinase-like protein SPH-1	3.0E-05	AMB36342.1	
			VVCAGIWQGGK		Nikobin	5.0E-06	E5AJX2.1	
**24a**	C	85	VVCAGIWQGGK	svSP	Nikobin	5.0E-06	E5AJX2.1	
**24b**	C	35	VVCAGIWQGGK	svSP	Nikobin	5.0E-06	E5AJX2.1	
			LMGWGTISTSK			6.0E-05	ABG26974.1	
**25a**	C	85	VVCAGIWQGGK	svSP	Nikobin	5.0E-06	E5AJX2.1	
			LMGWGTISTSK		thrombin-like enzyme	6.0E-05	O13069.1	

**25b**	C	35	VIGGDQCDINEHPFLAFVT DSR	svSP	Nikobin	4.0E-13	E5AJX2.1	
			1142.55-DAVLTAAHCNGK		Nikobin	3.0E-04	E5AJX2.1	
			LMGWGTISSTK		Cadam10_SVSP-11	6.0E-05	JAV48393.1	
**26a**	C	100	VVCAGIWQGGK	svSP	Nikobin	5.0E-06	E5AJX2.1	
			LMGWGTISSTK		Cadam10_SVSP-11	6.0E-05	JAV48393.1	
**26b**	C	40	PYGWGTISSTK	svSP	Cadam10_SVSP-11	3.0E-03	JAV48393.1	
			VSTLNEHPFLA		Vipera russelli proteinase RVV-V homolog 1	6.6E-02	P86530.1	
**27**	C	70	911.39-ESGECCDQCK	svMP	metalloproteinase	1.0E-05	ADI47577.1	
			CPLTLYQCR		group III metalloproteinase	7.0E-05	ADW54339.1	
**28**	C	35	AAYPWLLER	svSP	serine proteinase SP-3	1.0E-04	AMB36344.1	
			NPGVYTK		thrombin-like enzyme asperase	8.8E-02	Q072L6.1	
**29a**	C	65	414.2-NLNVVWNEQFLGCR		1-phosphatidylinositol 4,5-bisphosphate phosphodiesterase epsilon-1	8.0E-04	XP_015678180.1	
			HGVKELELELKSQL-343.23		phosphopantothenate-cysteine ligase	5.1E-02	APD70905.1	
**29b**	C	35	AAYPWLLER	svSP	serine proteinase SP-3	1.0E-04	AMB36344.1	
**29c**	A; C	13	GWGNVWIGLWGK	CTL	C-type lectin 1	2.0E-06	Q6T7B7.1	16108.34, 16208.30
			FGSWEWTDGSSTK			1.9E-02		
**30a**	C	60	NVEEGWYANLGPMR	LAAO	L-amino-acid oxidase	2.0E-08	G8XQX1.1	
			SAGQLYEELSKK			1.7E-02	BAN82140.1	
**30b**	C	40	NVEEGWYANLGPMR	LAAO	L-amino-acid oxidase	2.0E-08	G8XQX1.1	
			560.25-AQNMDQLPTSMYR			7.0E-06	XP_015673892.1	
			VTVLEASER			3.0E-03	P56742.2	
			PYVKPSEEGK			4.2E-02		
			SAGQLYEESLKK		BATXLAAO1	3.0E-06	JAV01888.1	
**30c**	C	20	MGWYVWIGLWNQR	CTL	C-type lectin-like protein 3A	2.0E-06	AJO70726.1	
			CGDDYPFVCK			6.0E-06		
			WTDGSSVIYK			3.0E-05		

**30d**	C	13	GSHLVSHLNIAEADFVVKK	CTL	snaclec-8	2.0E-09	APB93445.1
			AWNEGINCFVFK			4.0E-07	
			830.45-NLAEEGFVVKK		C-type lectin 5	7.0E-03	Q4PRC8.1
**31a**	C	65	VTVLEASER	LAAO	L-amino-acid oxidase	3.0E-03	P56742.2
**31b**	C	20	CGDDYPFVCK	CTL	C-type lectin-like protein 3A	6.0E-06	AJO70726.1
**32a**	C	60	FNVCCGRFYGECCDQCR	DI	disintegrin isoform Dc-1	1.0E-04	AAY43684.1
			SVGIVEDYR	svMP	metalloproteinase	8.0E-04	ADI47619.1
**32b**	C	35	374.16-YVWLGLWNQR	CTL	snaclec 2	1.0E-05	Q6T7B6.1
			WTDGSSVLYK		C-type lectin-like protein 3A	7.0E-04	AJO70726.1
**32c**	C	20	-	-	unknown (protein)	-	-
**33a**	C	65	YNYSKDPDFGMVDHGTK	svMP	Cadam10_SVMPIII-1	1.0E-09	JAV48413.1
			SVGIVEDYR		metalloproteinase	8.0E-04	ADI47619.1
			CEYWVSPANPGECCDQCR		metalloprotease 3	1.0E-03	AAR19274.1
**33b**	C	35	AAYPWLLER	svSP	serine proteinase SP-3	1.0E-04	AMB36344.1
**33c**	C	20	-	-	unknown (protein)	-	-
**34a**	C	65	SSVGLIQDYCK	svMP	metalloproteinase H4-A	7.0E-06	AHB62069.1
			PDYAMVDLGTK		metalloproteinase MP-2	5.0E-05	AAX86634.1
**34b**	C	35	NVEEGWYANLGPMR	LAAO	L-amino-acid oxidase	2.0E-08	G8XQX1.1
			VTVLEASER			3.0E-03	P56742.2
			SAGQLYEESLKK		BATXLAAO1	3.0E-06	JAV01888.1
			LYEMVNTLNMVFR	svMP	MPIII-1	1.0E-06	AMB36352.1
			SDPDYAMVDLGTK		metalloproteinase MP-2	5.0E-06	AAX86634.1
			SSVGLIQDYCK		metalloproteinase H4-A	7.0E-06	AHB62069.1
**34c**	C	20	NPQCLLNQPLR	svMP	metalloproteinase	3.0E-05	ADI47711.1
			AAYPWLLER	svSP	serine proteinase SP-3	1.0E-04	AMB36344.1
			LPSSPPSVGSV-330.16		transmembrane protease serine 9-like	1.0E-04	XP_015671564.1
**35a**	C	65	SDPDYAMVDLGTK	svMP	metalloproteinase MP-2	4.0E-06	AAX86634.1
			LYEMVNTLNMVFR		metalloproteinase MPIII-1	1.0E-06	AMB36352.1
**36a**	C	60	SCIMSGTLTCEASIR	svMP	H3 metalloproteinase precursor 1	4.0E-09	AGL45259.1

			MPQCILNKPLK		metalloproteinase	9.0E-07	ADI47653.1	
			ASQLNLTPEQQR		group III metalloproteinase	1.0E-06	ADW54334.1	
			385.15-QIYYTPSDENK		Zinc metalloproteinase-disintegrin-like VLAIP-A	1.0E-06	Q4VM08.1	
			DCQQCPCNAATCK		BATXSVMPIII2	5.0E-04	JAV01862.1	
			LVLVADYIMFLK		Metalloproteinase (type III) 6a	2.0E-06	JAV51432.1	
			NVEEGWYANLGMPR	LAAO	L-amino-acid oxidase	1.0E-04	G8XQX1.1	
**36b**	C	35	AAYPWLLER	svSP	serine proteinase SP-3	1.0E-04	AMB36344.1	
			NPGVYTK			1.0E-01		
**-**	A	-	-	-	unknown (protein)	-	-	24499.87, 27652.96

Table S2. Venom proteins and peptides identified from *Vipera ammodytes montandoni*. Assignment of venomic components by crude venom intact mass profiling (method A) and bottom-up (method C). RP-HPLC fractions with low masses were additionally analyzed by intact mas profiling (method B). Peak numbers based on RP-HPLC (see Figure 4B) and TIC (see Figure 2B) annotation. Sequence tags were obtained de novo from MS/MS spectra and identified against a non-redundant *Viperidae* protein database (taxid: 8689) by BLASTP. SDS-PAGE and intact mass profile analysis provided the molecular weight. “-“

Peak Number	Method	SDS PAGE M [kDa]	Identification sequence tag	Protein species	Protein ID	BLAST E-value	NCBI accession number	intact mass m/z native avarage
**1**	A; B	-	-	-	unknown (peptide)	-	-	451.00, 484.21, 506.19, 612.27, 686.99, 1801.00, 2229.00, 6842.00, 7384.28
**2**	A; B	-	DNEPPKKVPPN	svMP-i	endogenous tripeptide metalloproteinase inhibitor precursor	3.0E-06	AMB36336.1	496.24, 688.36, 1234.65
**3**	A; B	-	pEKW	svMP-i	svMP-i tripeptide	-	-	444.22, 466.21, 887.44, 963.34
**4**	A; B	-	pEKW	svMP-i	svMP-i tripeptide	-	-	444.23, 460.22, 754.36, 814.36
**5**	A; B	-	pEKW	svMP-i	svMP-i tripeptide	-	-	444.22, 452.15, 466.21, 468.12, 719.40, 723.36, 747.40, 809.39, 887.44, 963.34, 1072.60, 1110.54, 7384.27, 7531.52
		-	GGGGGGW	svMP-i	endogenous tripeptide metalloproteinase inhibitor precursor	4.0E-01	AMB36336.1	

**6**	A; B	-	pENW	svMP-i	svMP-i tripeptide	-	-	430.17, 859.34, 1072.6, 1288.51
**7**	A; B	-	-	-	unknown (peptide)	-	-	470.72, 1072.60
**8**	A; B	-	PEGPPLMEPHE	svMP-i	endogenous tripeptide metalloproteinase inhibitor precursor	9.0E-07	AMB36336.1	451.31, 2854.40, 2951.45, 3501.81, 3930.96, 5776.60, 6842.05, 7384.32, 7375.43
**9**	A; B	-	-	-	unknown (peptide)	-	-	416.14, 456.18, 584.28, 699.31, 836.37, 3664.67, 3761.72, 4176.86, 4214.80
**10**	A; B	-	-	-	unknown (peptide)	-	-	456.22, 722.37, 759.74, 833.44, 904.51, 932.51, 1144.63, 1182.58, 3283.50, 3381.55, 3834.62, 3872.59, 3928.99, 4174.87
**11**	A; B	-	-	-	unknown (peptide)	-	-	608.30, 681.30, 722.38, 833.44, 932.51, 1006.55, 1144.63, 1182.58, 1460.80, 3796.73, 4730.20, 13971.71
**12**	A; B	-	-	-	unknown (peptide)	-	-	932.52, 1144.62
**13**	A; B	-	-	-	unknown (peptide)	-	-	932.52, 1144.62, 1314.73
**14**	A; B	-	-	-	unknown (peptide)	-	-	573.03, 744.13, 1102.59
**15**	A; B	-	-	-	unknown (peptide)	-	-	-
**16**	A; C	14	PFLEVHER	VEGF-F	snake venom vascular endothelial growth factor	3.0E-03	P67863.2	834.39, 947.48, 1159.59
**17**	A; C	14	PFLEVHER	VEGF-F	snake venom vascular endothelial growth factor	3.0E-03	P67863.2	834.39, 947.48, 1159.59
**18a**	A; C	25	-	-	unknown (protein)	-	-	21199.58, 21298.85
**18b**	A; C	14	-	-	unknown (protein)	-	-	16307.27
**19**	A; C	-	-	-	unknown (protein)	-	-	13553.82, 13590.75
**20**	A; C	13	152.12-FFVHDCCYGR	PLA_2	Ammodytin I1(A) variant	2.0E-05	CAE47140.1	3532.76, 13890.28, 13988.22
			LAIYYYSFK		Vipoxin Chain B	2.0E-04	1AOK_B	

			NLFQFAK		Vaspin basic subunit variant	7.2E-02	CAE47300.1	
**21a**	A; C	23	310.13-YVCQYCPAGNLIGK	CRISP	cysteine-rich venom protein	6.0E-09	P79845.2	24654.40, 24750.41
			SVDFDSESPR			2.0E-05	P86537.1	
			MEWYPEAAAWQGV			1.0E-04	BAP39957.1	
			CWNLLMSPYPMK			1.8E-02	Q7ZZN9.1	
**21b**	C	13	NLFQFAK	PLA_2	Vaspin basic subunit variant	7.2E-02	CAE47300.1	-
**22**	A; C	23	MEWYPEAAANAWV	CRISP	cysteine-rich venom protein	5.0E-07	BAP39957.1	24547.04
			LVRAECGENLYMSTSMPK			7.0E-05	AMB36337.1	
			260.09-WYPEAAANSLR			3.0E-03	XP_015678374.1	
			SSLSNHLVKVDLHNSLR			6.0E-02	CE73575.1	
**23a**	C	21	YGCYCGWGGQGR	PLA_2	phospholipase A2 I acidic chain	3.0E-08	A60512	-
			RGLCLGENVNTYDK		Vaspin acidic subunit (1)	4.0E-06	CAE47105.1	
			239.13-FQFGDMILQK		Vaspin acidic subunit (1)	1.0E-05		
**23b**	A; C	13	241.16-LSSFGENMNTYDK	PLA_2	ammodytin I1(C) variant	1.0E-05	CAE47172.1	13624.69
			262.15-SQFGDMINK			3.0E-04		
			362.17-NGDIVCDNDAKER		Vaspin acidic subunit (1) variant	2.1E-02	CAE47105.1	
**24a**	C	21	GRLCLGENVNTYDK	PLA_2	acidic phospholipase A2 inhibitor chain HPD-1	4.0E-06	A4VBF0.1	
**24b**	A; C	13	LGAICFGENMNTYSR	PLA_2	Ammodytin I1(A) variant	2.0E-07	CAE47141.1	13676.81
			VAAMRFGENMNTYDK		Ammodytin I1(C) variant	7.0E-07	CAE47172.1	
			262.15-SQFGDMINK			3.0E-04		
			201.99-TFVHDCCYGR		Vaspin basic subunit variant	5.0E-05	CAE47300.1	
			362.15-NGDIVCGGDD-526.27		phospholipase A2	9.0E-05	AHJ09559.1	
**25a**	C	37	541.21-GTLLNQEWVLTAAR	svSP	venom serine proteinase-like protein 2	1.0E-07	Q9PT40.1	
			TLCAGLLQGGLDSSSK			3.0E-04		
			LMGWGTLTTTK			6.0E-04		
**25b**	C	23	-	-	unknown (protein)	-	-	
**25c**	C	15	KAICFGENMNTYSR	PLA_2	Ammodytin I1(A) variant	2.0E-07	CAE47141.1	
**26a**	C	>200	-	-	unknown (protein)	-	-	
**26b**	C	60	-	-	unknown (protein)	-	-	
**26c**	A; C	37	LVNDECNINEHPFLAFVPPHQ	svSP	Nikobin	1.0E-09	E5AJX2.1	32026.88, 32899.08

			409.24-QPGLYTDIFDY-629.33			9.0E-07		
			VVCAGIWQGGK			5.0E-06		
			WKPPVVGSVCR			2.0E-03		
**26d**	C	15	-	-	unknown (protein)	-	-	
**27**	A; C	37	409.24-KPGLYTDIFDYS-629.32	svSP	Nikobin	2.0E-06	E5AJX2.1	32686.16, 35124.93
			WKPPVVGSVCR			2.0E-03		
			LMGWGTISSTK		Cadam10_SVSP-11	6.0E-05	JAV48393.1	
**28a**	A; C	37	PDNHLFAYNEHPFLAFVTSDR	svSP	Nikobin	1.0E-07	E5AJX2.1	32686.34, 33342.02
			VVCAGIWQGGK			5.0E-06		
**28b**	C	25	MEWYPEAAANPMK	CRISP	cysteine-rich venom protein	7.0E-06	XP_015678374.1	
			838.43-LLDLHNSLR			1.6E-01		
**28c**	C	15	-	-	unknown (protein)	-	-	
**29a**	A; C	60	LLTAIDFQRTLGK	svMP	metalloproteinase type III	8.0E-05	JAS05425.1	24547.96, 27654.98
			997.58-LTAIDFNGLTKK			3.0E-04	ADI47577.1	
			AYIGTMCQPK			4.0E-04	ADW54356.1	
**29b**	C	37	-	-	unknown (protein)	-	-	
**29c**	C	25	1098.52-TLNNDIMLLK	other	trypsin-like	7.0E-04	XP_015672094.1	
**29d**	C	15	1098.52-TLNNDIMLLK	other	trypsin-like	7.0E-04	XP_015672094.1	
**30a**	C	55	326.20-LNEMYLPLNLR	svMP	metalloproteinase	8.0E-05	ADI47590.1	
**30b**	C	37	VVCAGIWQGGK	svSP	Nikobin	5.0E-06	E5AJX2.1	
**30c**	C	30	AAYPWLLER	svSP	serine proteinase SP-3	1.0E-04	AMB36344.1	
**30d**	A; C	13	287.13-DDAEMFCR	CTL	C-type lectin	5.0E-03	Q6T7B7.1	13890.25
			204.07-SWEWTDGSSTK			1.3E-02		
			331.12-TEFAEYLADYLK		C-type lectin mannose-binding isoform-like	3.6E-02	XP_015686729.1	
**31a**	C	55	-	-	unknown (protein)	-	-	
**31b**	C	37	LYDYSVCR	svSP	serine protease	1.0E-02	ADI47570.1	
**31c**	C	30	AAYPWLLER	svSP	serine proteinase SP-3	1.0E-04	AMB36344.1	
**32a**	C	37	-	-	unknown (protein)	-	-	
**32b**	C	30	AAYPWLLER	svSP	serine proteinase SP-3	1.0E-04	AMB36344.1	
**33a**	C	55	HDDIFAYEK	LAAO	L-amino-acid oxidase	1.0E-04	Q6WP39.1	

			VTVLEASER			3.0E-03	JAS05318.1
**33b**	C	50	HDDIFAYEK	LAAO	L-amino-acid oxidase	1.0E-04	Q6WP39.1
**33c**	C	37	-	-	unknown (protein)	-	-
**33d**	C	30	AAYPWLLER	svSP	serine proteinase SP-3	1.0E-04	AMB36344.1
**33e**	C	20	WTDGSSVIYK	CTL	C-type lectin-like protein 3A	3.0E-05	AJO70726.1
			537.22-VWLGLWELR			7.4E-02	
**33f**	C	11	EKNEGINCFVFEIAK	CTL	snaclec-8	2.0E-08	APB93445.1
**34a**	C	50	EDDYEEFLEIAK	LAAO	L-amino-acid oxidase	1.0E-07	G8XQX1.1
			213.12-EEGWYANLGNNR			3.0E-04	
			HDDIFAYEK			1.0E-04	Q6WP39.1
			523.22-FSEALTAPEGR			3.0E-04	P0DI84.1
			158.07-GQLYEESLK		BATXLAAO1	1.0E-03	JAV01888.1
**34b**	C	37	HDDIFAYEK	LAAO	L-amino acid oxidase 1b	1.0E-04	Q6WP39.1
**35a**	C	50	VTVLEASER	LAAO	L-amino acid oxidase 1b	3.0E-03	JAS05318.1
			523.22-FSEALTAPWR			4.7E-02	
**35b**	C	37	-	-	unknown (protein)	-	-
**35c**	C	30	AAYPWLLER	svSP	serine proteinase SP-3	1.0E-04	AMB36344.1
**35d**	C	20	537.21-VWLGLWELR	CTL	C-type lectin galatrox	8.1E-02	P0DM53.1
**35e**	C	11	371.17-EGINCFVFEIAK	CTL	snaclec-8	2.0E-07	APB93445.1
**36a**	C	70	VEDYDQIGASLR	other	xaa-Pro aminopeptidase 2	6.0E-06	XP_015676063.1
			414.22-FMGSTWQEK			7.0E-05	
			LEDVALVVPAK			2.0E-03	
**36b**	C	50	SSVGLIQDYCK	svMP	metalloproteinase H4-A	7.0E-06	AHB62069.1
			HDDIFAYEK	LAAO	L-amino-acid oxidase	1.0E-04	Q6WP39.1
			VTVLEASER			3.0E-03	JAS04872.1
			523.22-FSEALTAPEGR			3.0E-04	P0DI84.1
**36c**	C	30	-	-	unknown (protein)	-	-
**37a**	C	50	SSVGLIQDYCK	svMP	metalloproteinase H4-A	7.0E-06	AHB62069.1
			HDDIFAYEK	LAAO	L-amino-acid oxidase	1.0E-04	Q6WP39.1
			VTVLEASER			3.0E-03	JAS04872.1

**37b**	C	30	NPQCILNQLPR	svMP	metalloproteinase	4.0E-03	ADI47711.1	
			1199.58-LTLELMLLK	other	ATP synthase F0 subunit 8	7.5E-02	AMW93172.1	
**38a**	C	50	247.16-LMSGTLSCEASIR	svMP	metalloproteinase precursor 1	1.0E-06	AGL45259.1	
			NPCQIYYTPR			3.0E-06		
**38b**	C	37	-	-	unknown (protein)	-	-	
**38c**	C	30	-	-	unknown (protein)	-	-	
**X**	A	-	-	-	unknown (protein)	-	-	13890.25, 27654.95, 27801.99

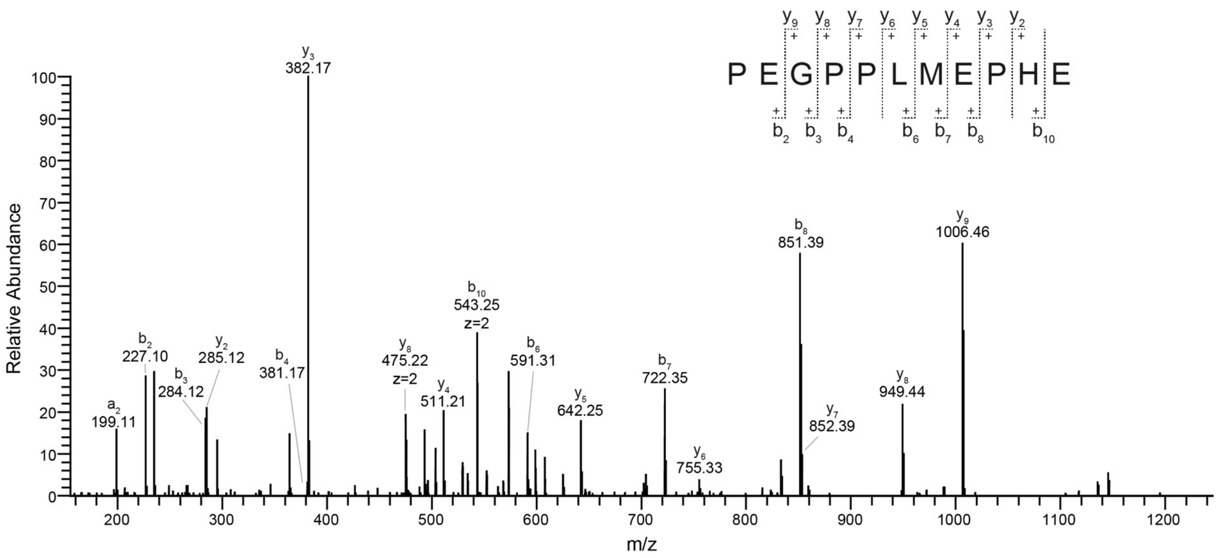

Figure S1. Tripeptide precursor fragment of *V. a. transcaucasiana* peak 9. The annotated MS1 spectrum of Vat by intact mass profiling peak 9 at RT 1.74 min shows the mass of the tripeptide precursor fragment PEGPPLMEPHE.

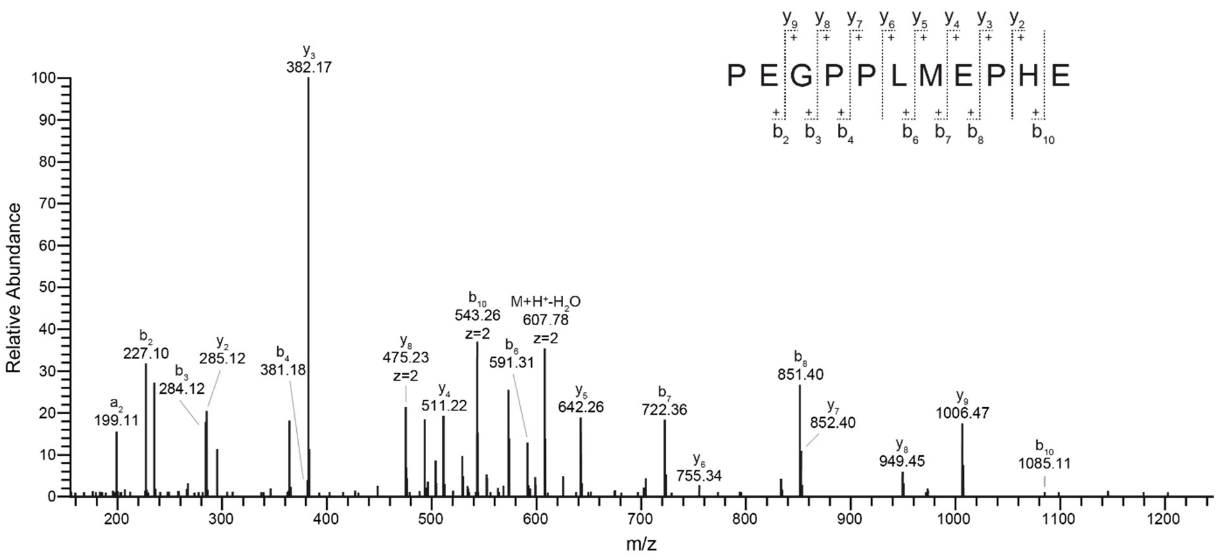

Figure S2. Tripeptide precursor fragment of *V. a. montandoni* peak 8. The annotated MS1 spectrum of Vam by intact mass profiling peak 8 at RT 1.90 min shows the mass of the tripeptide precursor fragment PEGPPLMEPHE.

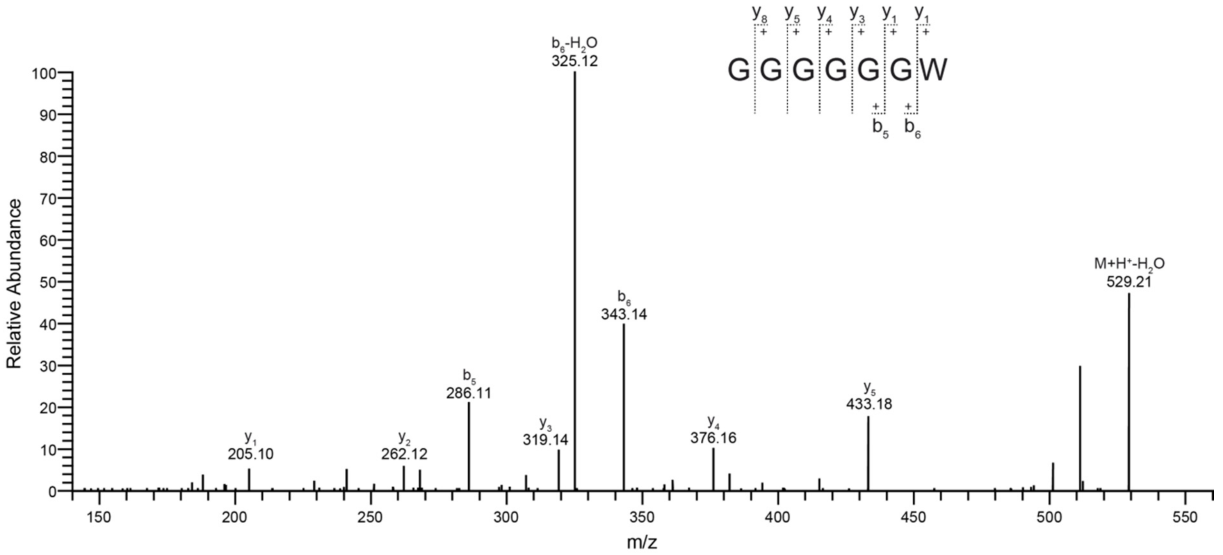

Figure S3. Tripeptide precursor fragment of *V. a. transcaucasiana* peak 4. The annotated MS1 spectrum of Vat by intact mass profiling peak 4 at RT 2.28 min shows the mass of the tripeptide precursor fragment GGGGGGW.

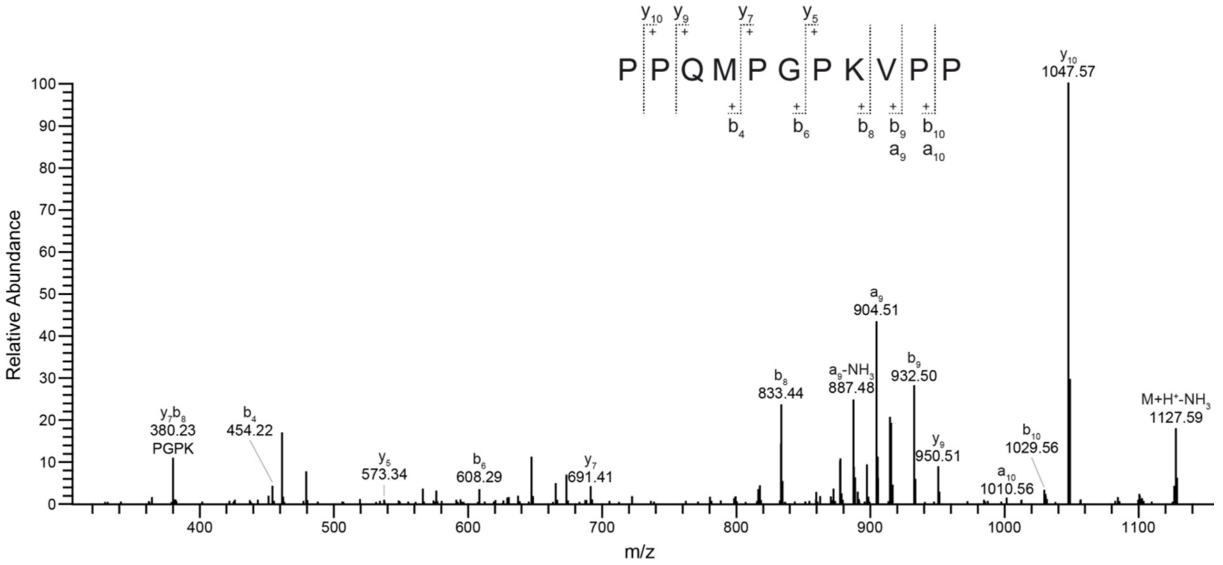

Figure S4. Tripeptide precursor fragment of *V. a. transcaucasiana* peak 14. The annotated MS1 spectrum of Vat by intact mass profiling peak 14 at RT 1.96 min shows the mass of the tripeptide precursor fragment PPQMPGPKVPP.

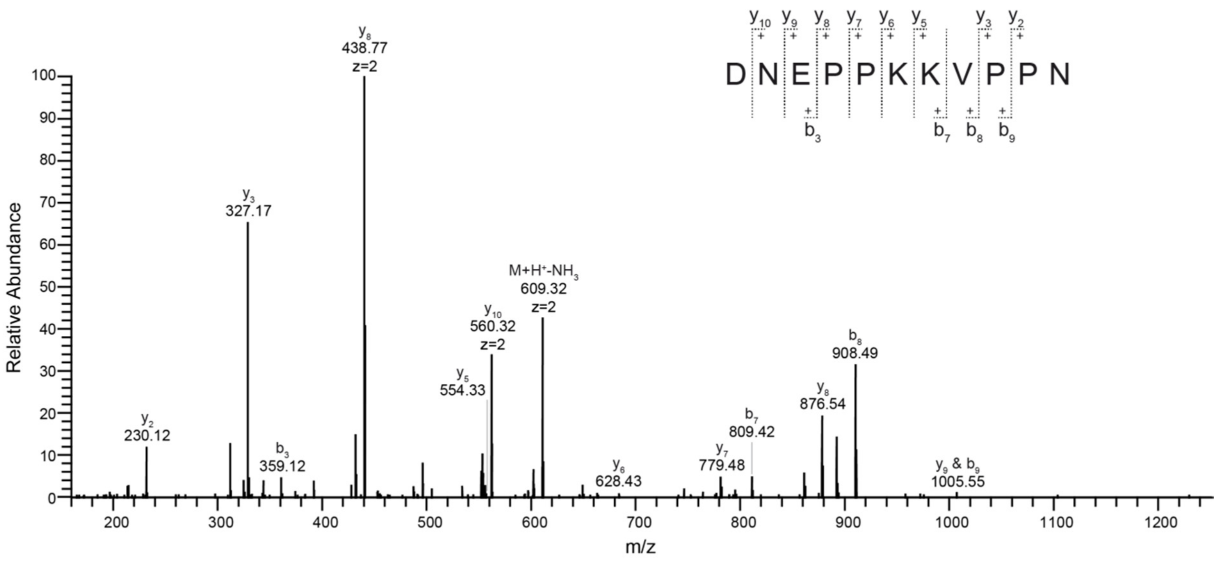

Figure S5. Tripeptide precursor fragment of *V. a. montandoni* peak 2. The annotated MS1 spectrum of Vam by intact mass profiling peak 2 at RT 1.57 min shows the mass of the tripeptide precursor fragment DNEPPKKVPPN.

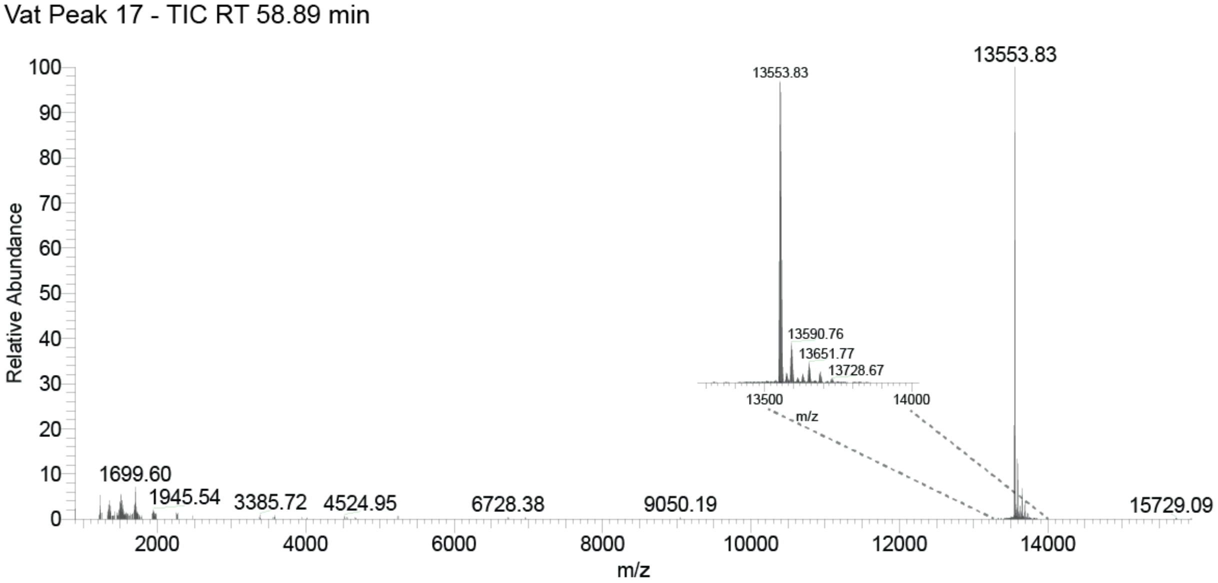

Figure S6. Deconvoluted masses of *V. a. transcaucasiana* peak 17. The deconvoluted MS1 spectrum of Vat intact mass profiling peak 17 at RT 58.89 min shows the mass of the neutral phospholipase A_2 Ammodytin I2.

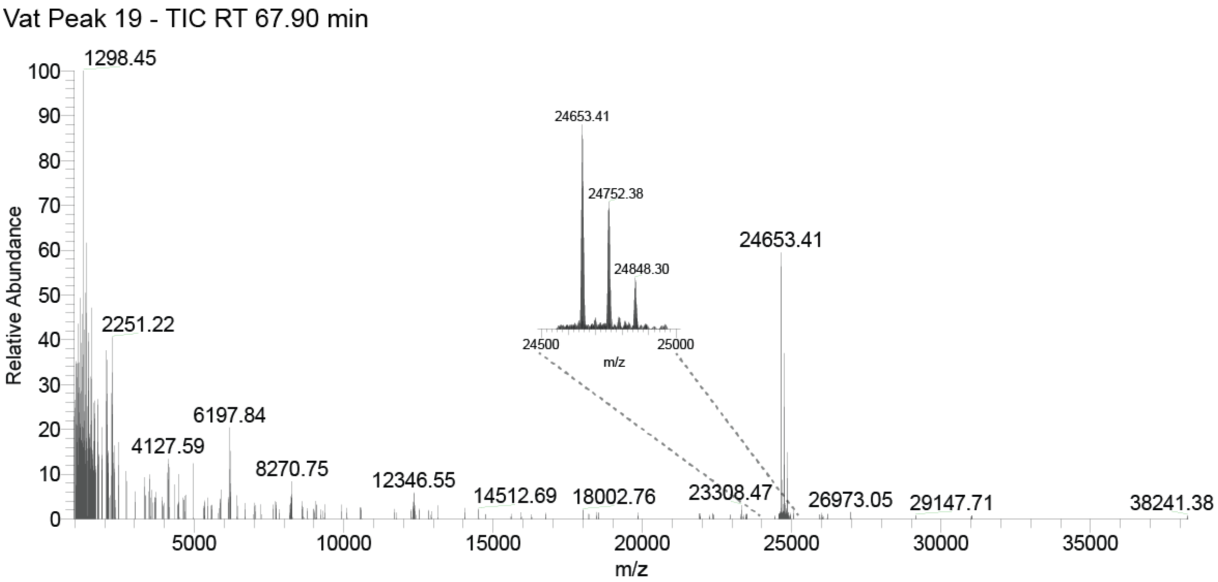

Figure S7. Deconvoluted masses of *V. a. transcaucasiana* peak 19. The deconvoluted MS1 spectrum intact mass profiling peak 19 at RT 67.91 min shows the masses of CRISP's.

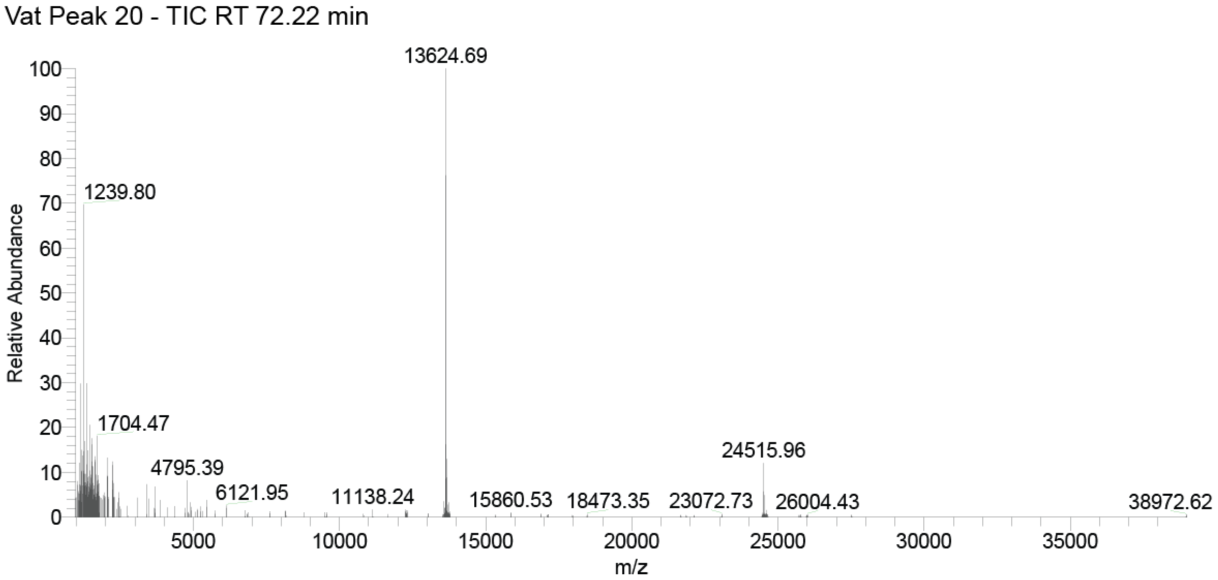

Figure S8. Deconvoluted masses of *V. a. transcaucasiana* peak 20. The deconvoluted MS1 spectrum of Vat intact mass profiling peak 20 at RT 72.22 min shows the mass closely related to the acidic phospholipase A_2 homolog Vipoxin A chain.

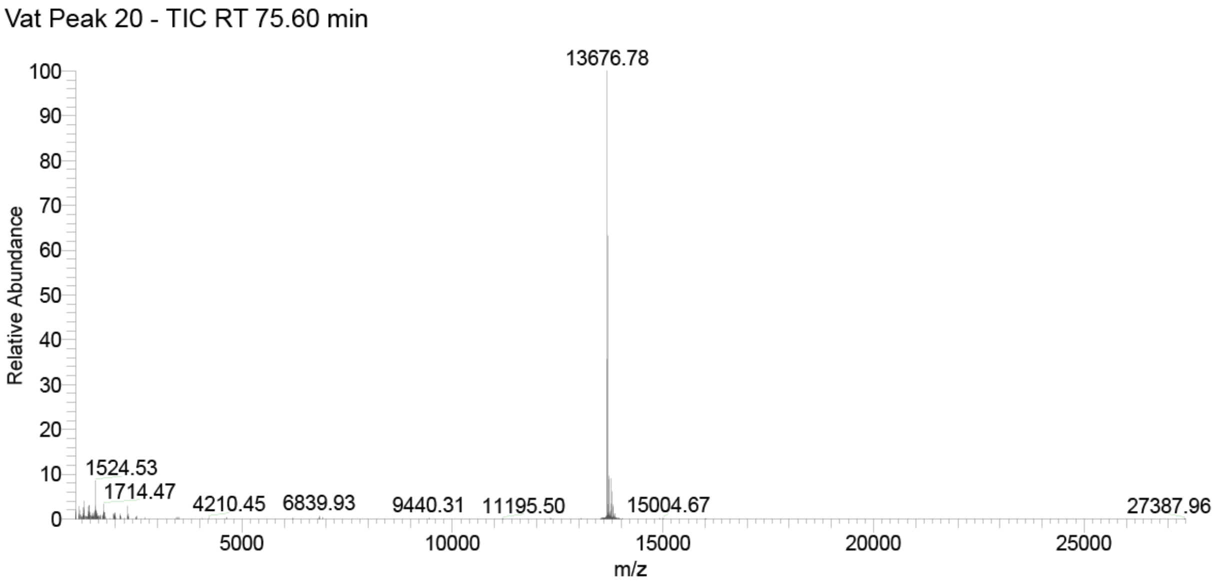

Figure S9. Deconvoluted masses of *V. a. transcaucasiana* peak 20. The deconvoluted MS1 spectrum of Vat intact mass profiling peak 20 at RT 75.60 min shows the mass of a PLA_2.

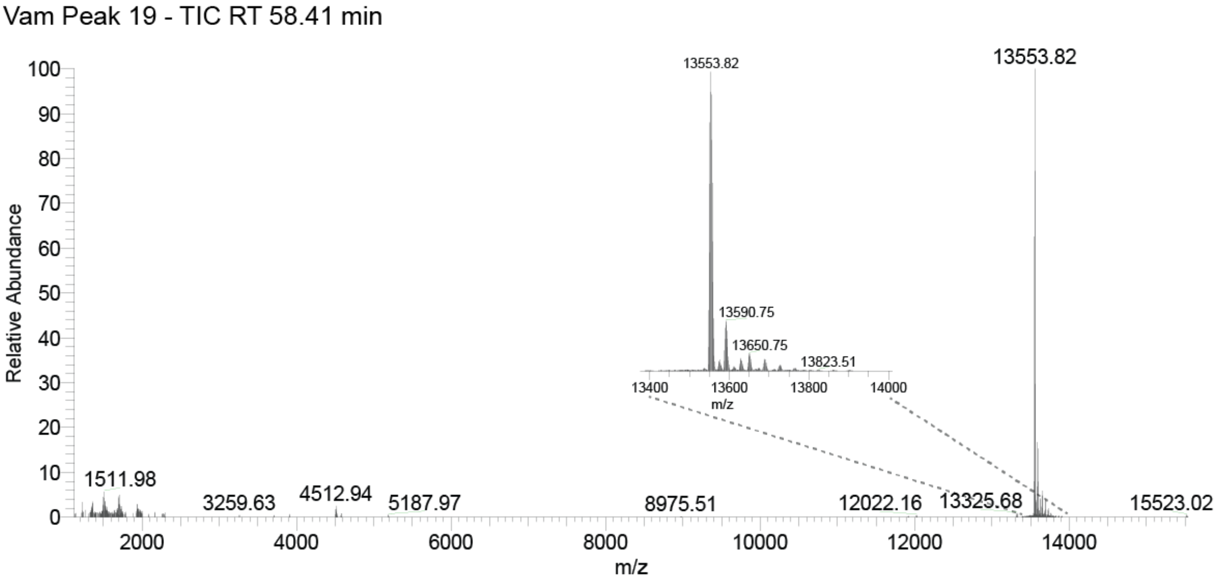

Figure S10. Deconvoluted masses of *V. a. montandoni* peak 19. The deconvoluted MS1 spectrum of Vam intact mass profiling peak 19 at RT 58.41 min shows the mass of the neutral phospholipase A_2 Ammodytin I2.

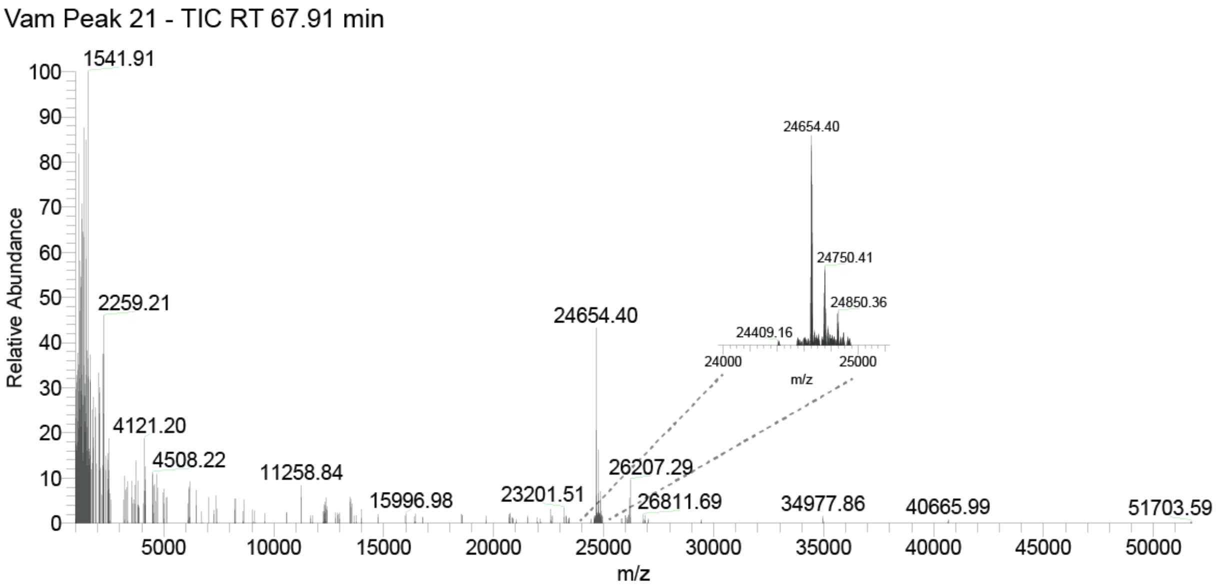

Figure S11. Deconvoluted masses of *V. a. montandoni* peak 21. The deconvoluted MS1 spectrum of Vam intact mass profiling peak 21 at RT 67.91 min shows the mass of CRISP's.

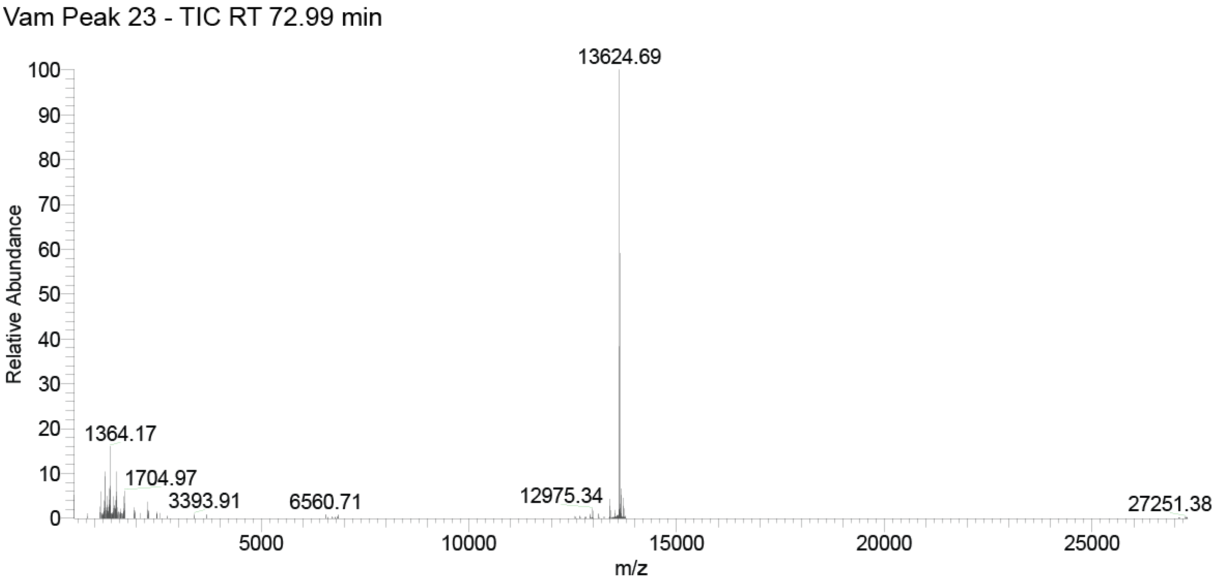

Figure S12 Deconvoluted masses of *V. a. montandoni* peak 23. The deconvoluted MS1 spectrum of Vam intact mass profiling peak 23 at RT 72.99 min shows the mass closely related to the acidic phospholipase A_2 homolog Vipoxin A chain.

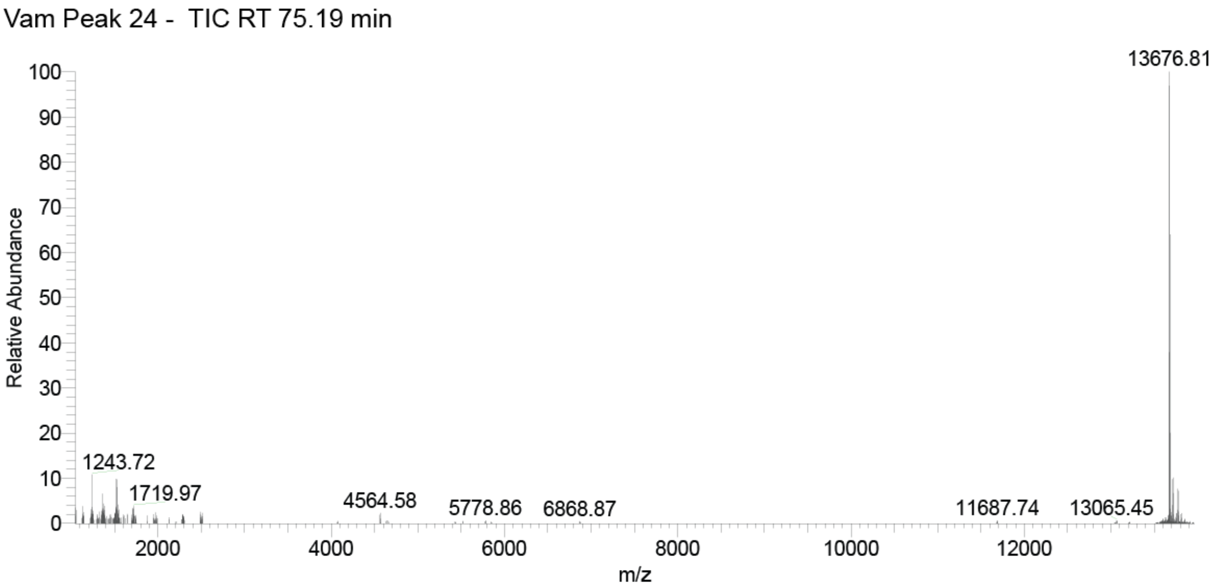

Figure S13. Deconvoluted masses of *V. a. montandoni* peak 24. The deconvoluted MS1 spectrum of Vam intact mass profiling peak 24 at RT 75.19 min shows the mass of a PLA_2.

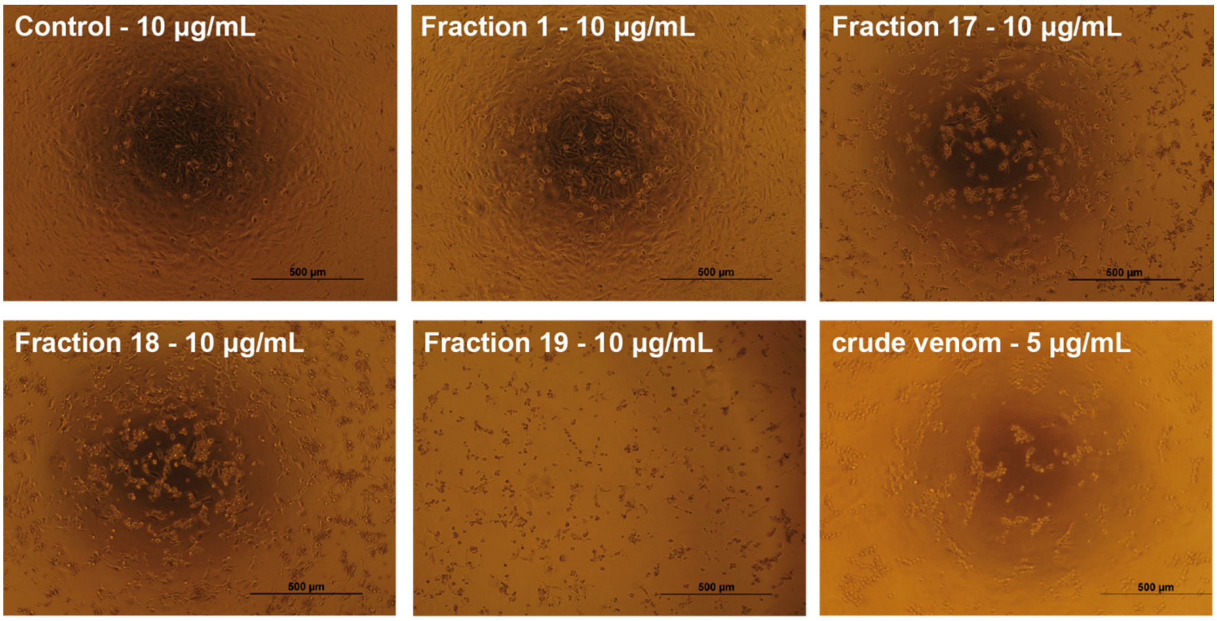

Figure S14. Cytotoxicity analysis of fractionated components from *Vipera a. transcaucasiana*. The *in vitro* cytotoxicity testing with crude venoms and fractions was performed according to the protocol of Nalbantsoy and Hempel *et al*. [14]. Cytotoxicity assay was performed with crude and fractionated venom components for *Vipera a. transcaucasiana*. Cell viability was determined by MTT assay and control was exposed to vehicle only which was taken as 100 % viability. We tested all fractions against most sensitive cells MDA-MB-231 according to the screening of crude venom. The fractions with strongest cytotoxic effect on the triple negative MDA-MB-231 breast cancer cells are shown above.
